# Immunity to LuloHya and Lundep, the salivary spreading factors from *Lutzomyia longipalpis*, protects against *Leishmania major* infection

**DOI:** 10.1371/journal.ppat.1007006

**Published:** 2018-05-03

**Authors:** Ines Martin-Martin, Andrezza Campos Chagas, Anderson B. Guimaraes-Costa, Laura Amo, Fabiano Oliveira, Ian N. Moore, Thiago S. DeSouza-Vieira, Elda E. Sanchez, Montamas Suntravat, Jesus G. Valenzuela, Jose M. C. Ribeiro, Eric Calvo

**Affiliations:** 1 Laboratory of Malaria and Vector Research, National Institute of Allergy and Infectious Diseases, National Institutes of Health, Rockville, Maryland, United States of America; 2 Laboratory of Immunogenetics, National Institute of Allergy and Infectious Diseases, National Institutes of Health, Rockville, Maryland, United States of America; 3 Infectious Disease Pathogenesis Section, Comparative Medicine Branch, National Institute of Allergy and Infectious Diseases, National Institutes of Health, Rockville, Maryland, United States of America; 4 National Natural Toxins Research Center and Department of Chemistry, Texas A&M University-Kingsville, Kingsville, Texas, United States of America; Uniformed Services University of the Health Sciences, UNITED STATES

## Abstract

Salivary components from disease vectors help arthropods to acquire blood and have been shown to enhance pathogen transmission in different model systems. Here we show that two salivary enzymes from *Lutzomyia longipalpis* have a synergist effect that facilitates a more efficient blood meal intake and diffusion of other sialome components. We have previously shown that Lundep, a highly active endonuclease, enhances parasite infection and prevent blood clotting by inhibiting the intrinsic pathway of coagulation. To investigate the physiological role of a salivary hyaluronidase in blood feeding we cloned and expressed a recombinant hyaluronidase from *Lu*. *longipalpis*. Recombinant hyaluronidase (LuloHya) was expressed in mammalian cells and biochemically characterized *in vitro*. Our study showed that expression of neutrophil CXC chemokines and colony stimulating factors were upregulated in HMVEC cells after incubation with LuloHya and Lundep. These results were confirmed by the acute hemorrhage, edema and inflammation in a dermal necrosis (dermonecrotic) assay involving a massive infiltration of leukocytes, especially neutrophils, in mice co-injected with hemorrhagic factor and these two salivary proteins. Moreover, flow cytometry results showed that LuloHya and Lundep promote neutrophil recruitment to the bite site that may serve as a vehicle for establishment of *Leishmania* infection. A vaccination experiment demonstrated that LuloHya and Lundep confer protective immunity against cutaneous leishmaniasis using the *Lu*. *longipalpis—Leishmania major* combination as a model. Animals (C57BL/6) immunized with LuloHya or Lundep showed minimal skin damage while lesions in control animals remained ulcerated. This protective immunity was abrogated when B-cell-deficient mice were used indicating that antibodies against both proteins play a significant role for disease protection. Rabbit-raised anti-LuloHya antibodies completely abrogated hyaluronidase activity *in vitro*. Moreover, *in vivo* experiments demonstrated that blocking LuloHya with specific antibodies interferes with sand fly blood feeding. This work highlights the relevance of vector salivary components in blood feeding and parasite transmission and further suggests the inclusion of these salivary proteins as components for an anti-*Leishmania* vaccine.

## Author summary

Blood-feeding is key to sand flies reproductive success and an important link in *Leishmania* spp. transmission. While the sand flies attempt to modify the bite site to enhance blood feeding success, the host’s ability to react to injury becomes compromised and could facilitate pathogen invasion. In our model, several proteins found in the saliva of the New World sand fly *Lutzomyia longipalpis* contribute to enhance *Leishmania major* pathogenesis. Among these secreted salivary molecules, endonucleases (Lundep) and hyaluronidases (LuloHya) increase parasite virulence by destroying the neutrophil traps and disrupting the integrity of the extracellular matrix, respectively, leading to the diffusion of other salivary components; allowing the *Leishmania* parasite to evade the host immune response and to cause an infection. Immunization against these molecules significantly reduces *L*. *major* infection in mice. These vaccine candidates are intended to disrupt a very early step in the *Leishmania* transmission event, the internalization of parasites in neutrophils (without being killed) and spread from the inoculation site. The work presented here highlights the relevance of vector-based vaccine development to disrupt vector-borne diseases transmission.

## Introduction

Leishmaniasis, a vector-borne parasitic disease, comprises several clinical manifestations ranging from skin sores to life-threating visceral diseases. The causative agents, *Leishmania* parasites, are transmitted to the vertebrate host by the bite of infected female phlebotomine sand flies (Diptera: Psychodidae) [1]. During blood feeding, sand fly saliva is deposited into the vertebrate host skin. It consists of a mixture of pharmacologically active compounds that work in a redundant way to counteract vertebrate platelet aggregation, blood coagulation, vasoconstriction and inflammation as an insect strategy for blood feeding success [2–4].

Sand fly saliva modifies the bite site environment and is known to play a major role in parasite transmission [5–9]. Several proteins found in the saliva of *Lutzomyia longipalpis*, the main vector of visceral leishmaniasis in the New World, contribute to enhance *Leishmania* pathogenesis. The salivary peptide Maxadilan, which was described in the early 1990s as the most potent known vasodilatory substance [10], exacerbates *Leishmania* infection [11]. Likewise, the salivary endonuclease Lundep acts as an anticoagulant, anti-inflammatory and destroys the neutrophil extracellular traps (NETs) resulting in an increased parasite survival [9]. The fact that antibodies against Lundep block the DNase activity of female salivary gland extract (SGE), opens the possibility of a potential use of Lundep as an anti-*Leishmania* vaccine.

Hyaluronidases are enzymes commonly found in snake and arthropod venoms, blood sucking hookworms or leeches [12, 13]. They cleave hyaluronic acid (HA), a major component of the extracellular matrix in vertebrates [14]. Hyaluronidases can cause loss of the extracellular matrix leading to the diffusion of other salivary and/or venom components [13, 15]. Hyaluronidases are present in saliva of sand flies and have been postulated as *Leishmania* virulence factors. Volfova and others [16] showed that inoculation of *Leishmania major* parasites with bovine hyaluronidase resulted in larger lesions when compared with parasites injected alone in a BALB/c mouse model. Although hyaluronidase activity has been described in the saliva of a variety of hematophagous insects [15–18] the mechanisms of salivary hyaluronidase pathogenesis and its role in blood feeding are not well understood.

In this work we characterized the hyaluronidase from *Lu*. *longipalpis* saliva (LuloHya) and determined its physiological role in reducing the time of a blood meal as an essential factor for the insect to succeed in hematophagy. Furthermore, we show that LuloHya and Lundep could act as the salivary spreading factors increasing dissemination of other salivary components at the bite site. Finally, we show that immunization against these molecules significantly reduce *L*. *major* infection in mice, highlighting them as candidates for an anti-*Leishmania* vaccine.

## Results

### *In silico* analysis of LuloHya

**Fig 1:**
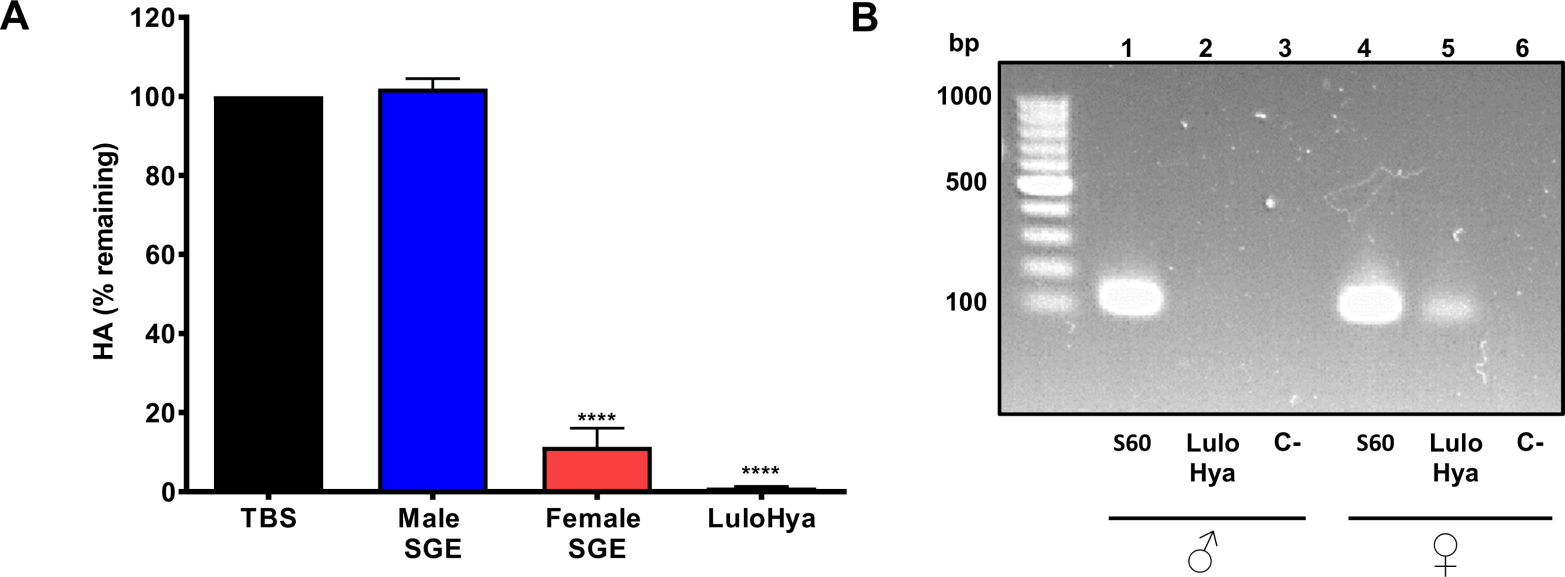
Characterization of LuloHya. **Hyaluronidase activity is female specific in *Lu*. *longipalpis*. (A)** SGE from 10 male *Lu*. *longipalpis* did not show any cleavage of HA in the turbidimetric assay, expressed as the % of remaining HA. On the contrary, SGE from 1 female and 10 nM recombinant LuloHya exhibited remarkable hyaluronidase activity. Multiple comparisons were done by one-way ANOVA (****: p<0.0001). Bars indicate the standard error of the mean (SEM). **(B)** Gene coding LuloHya is specifically expressed in female sand flies (lane 5). Sybr Safe 2% agarose gel electrophoresis of PCR products from RT-PCR of LuloHya gene with cDNA from male and female *Lu*. *longipalpis* head and thorax (lanes 2 and 5, respectively). S60 gene was used as the reference gene (lanes 1 and 4). Reaction mixes without DNA were included as negative controls (lanes 3 and 6). GeneRuler 1kb DNA ladder was used (Thermo Scientific).

**Fig 2:**
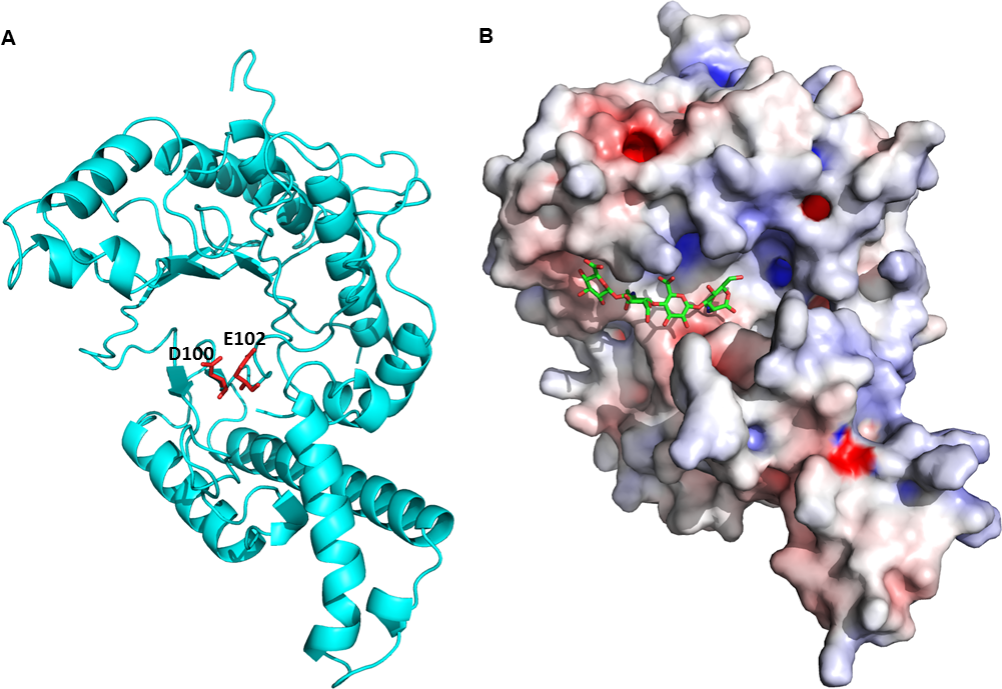
Three-dimensional modeling of LuloHya. Predicted 3D model of LuloHya was generated automatically by I-TASSER software. LuloHya coordinates used to generate the model were based on the ligand-free honeybee venom hyaluronidase (1FCQ) and in complex (1FCV) with hyaluronic acid tetramer. **(A)** Cartoon generated with PyMol shows LuloHya as a typical globular protein with a catalytic site topology of the cleft/groove class. Conserved amino acid residues aspartic acid (D100) and glutamic acid (E102) are highlighted in red. **(B)** Electrostatic potential of LuloHya generated by the APBS tools shows charged amino acids evenly distributed on the surface of the enzyme as determined by the Adaptive Poisson-Bolzmann Solver software with blue being positive and red being negative. Predicted substrate (HA tetramer) bound to LuloHya catalytic cleft if shown.

The presence of a putative salivary hyaluronidase in adult *Lu*. *longipalpis* was first described by bioinformatic survey of its sialotranscriptome [19]. Accordingly, hyaluronidase activity was found in SGE of female *Lu*. *longipalpis* but not in non-blood feeding male adult sand flies (Fig 1A). This sex specificity, at the protein and mRNA level (Fig 1B) suggests its involvement on blood feeding. Transcript AF132515 (LuloHya) codes for a putative hyaluronidase containing a signal peptide indicative of secretion with a calculated molecular weight (MW) of 42.28 kDa and pI of 7.98. The amount of LuloHya present in a salivary gland pair is 0.338 ng, which is in accordance with the previous estimated data derived from silver gel staining [20]. These authors suggested that the presence of a hyaluronidase in the salivary glands would be < 0.1% of the total salivary gland protein; If *Lu*. *longipalpis* SG protein content is 0.36 μg, the estimated hyaluronidase would be < 0.36 ng). LuloHya has 6 putative N-glycosylation sites in its primary amino acid sequence indicating that the native protein can be highly glycosylated. Alignment of LuloHya with other known hyaluronidases in the NCBI database reveals the presence of the conserved amino acids found in most hyaluronidase enzymes characterized so far [21]. The predicted 3D-structure of LuloHya (Fig 2) was generated automatically by I-TASSER software [22]. LuloHya coordinates used to generate the model were based on the ligand-free honeybee venom hyaluronidase (1FCQ) and in complex (1FCV) with hyaluronic acid tetramer [23]. The PyMol-generated model of LuloHya shows a typical globular protein structure resembling the classic (β/α)8 TIM barrel commonly found in hydrolases and present in 10% of all enzymes [24]. The predicted topology of the active site appears to fall into the cleft or groove class, which can accommodate polymeric substrates such as HA. The modeled catalytic site of LuloHya contains the conserved amino acid residues aspartic acid (D100) and glutamic acid (E102) which are likely involved in the catalytic activity as well as two arginine residues (R105 and R245) situated at opposite walls of the catalytic cleft (Fig 2). These 2 arginine residues may guide the HA into the active site through electrostatic interactions with the substrate [23].

### Biochemical characterization of LuloHya

**Fig 3:**
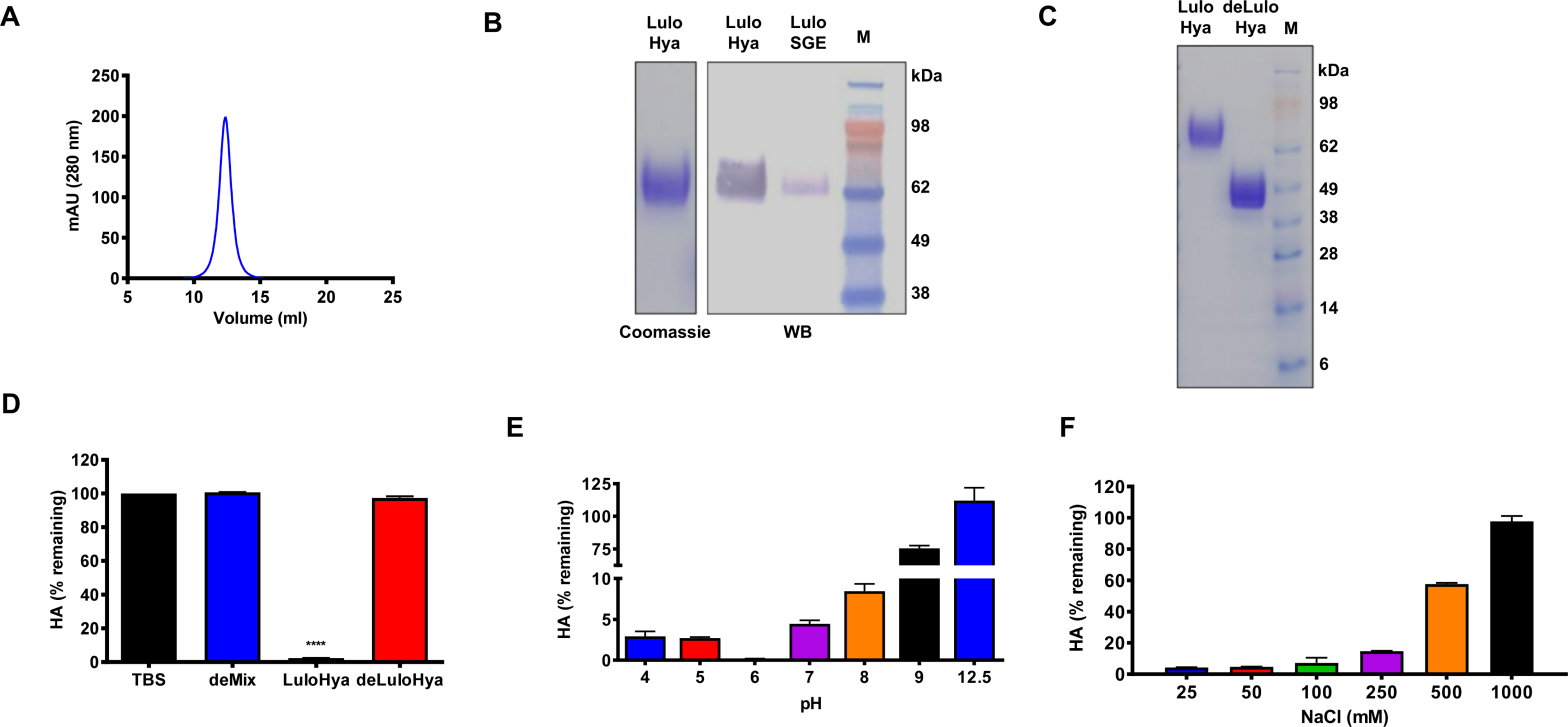
Biochemical characterization of the recombinant protein LuloHya. **(A)** Purification of LuloHya by size exclusion chromatography using Superdex 200 Increase 10/300 GL column. **(B)** Coomassie-stained gel electrophoresis of LuloHya (1 μg). Mouse anti-LuloHya antibodies (1:5,000) recognized LuloHya (100 ng) and a single band in the SGE (5 pairs of SG) by Western blot. M: SeeBlue Plus2 Pre-Stained Protein Standard (Life Technologies). **(C)** NuPAGE Novex 4–12% Bis-Tris protein gel shows differences in electrophoretic pattern of LuloHya (1 μg) and its deglycosylated form (deLuloHya: 1 μg) which runs at the expected molecular weight (42.3 kDa). M: SeeBlue Plus2 Pre-Stained Protein Standard (Life Technologies). **(D)** Hyaluronidase activity of 10 nM LuloHya and its deglycosylated form (deLuloHya). As negative controls, 4 micrograms of HA were incubated with TBS instead of recombinant protein or the deglycosylation enzyme mix (DeglycoMx Kit, QABio) without protein. **(E)** Turbidimetric assay showed a clear pH dependence of hyaluronidase activity of LuloHya. Reaction mixtures were prepared with solutions containing 25 mM buffer (described in Methods), 100 mM NaCl, 0.1% BSA, and different pH values (4–12.5; adjusted with a pHmeter 430, Corning). **(F)** Ionic strength dependency was analyzed in reaction mixtures of 25 mM HEPES, 0.1% BSA, pH 7.3 with variable NaCl concentration (25–1000 mM). Hyaluronidase activity is inversely expressed as the remaining HA (%) after treatment of 4 μg of HA with 10 nM enzyme during 1 h at 37°C. Biological triplicates and technical duplicates were assessed. Multiple comparisons were done by one-way ANOVA (****: p<0.0001). Bars indicate SEM.

To further characterize the catalytic and biological activity of this salivary enzyme, recombinant LuloHya was expressed in HEK cells and purified by affinity and size exclusion chromatography and visualized as a single band by Coomassie-staining gel electrophoresis (Fig 3A and 3B). Identity of the purified recombinant protein was confirmed by N-terminal sequencing. Mouse polyclonal anti-LuloHya antibodies specifically recognized LuloHya and a single band in the SGE, both running at an apparent MW of 70 kDa, higher than the calculated MW of 42 kDa (Fig 3B). This shift in electrophoretic mobility properties is due to the presence of sugar residues as *in vitro* deglycosylation of the recombinant protein recovers its expected MW under reducing conditions (Fig 3C). Moreover, glycosylation of LuloHya is critical for its enzymatic activity (Fig 3D).

**Fig 4:**
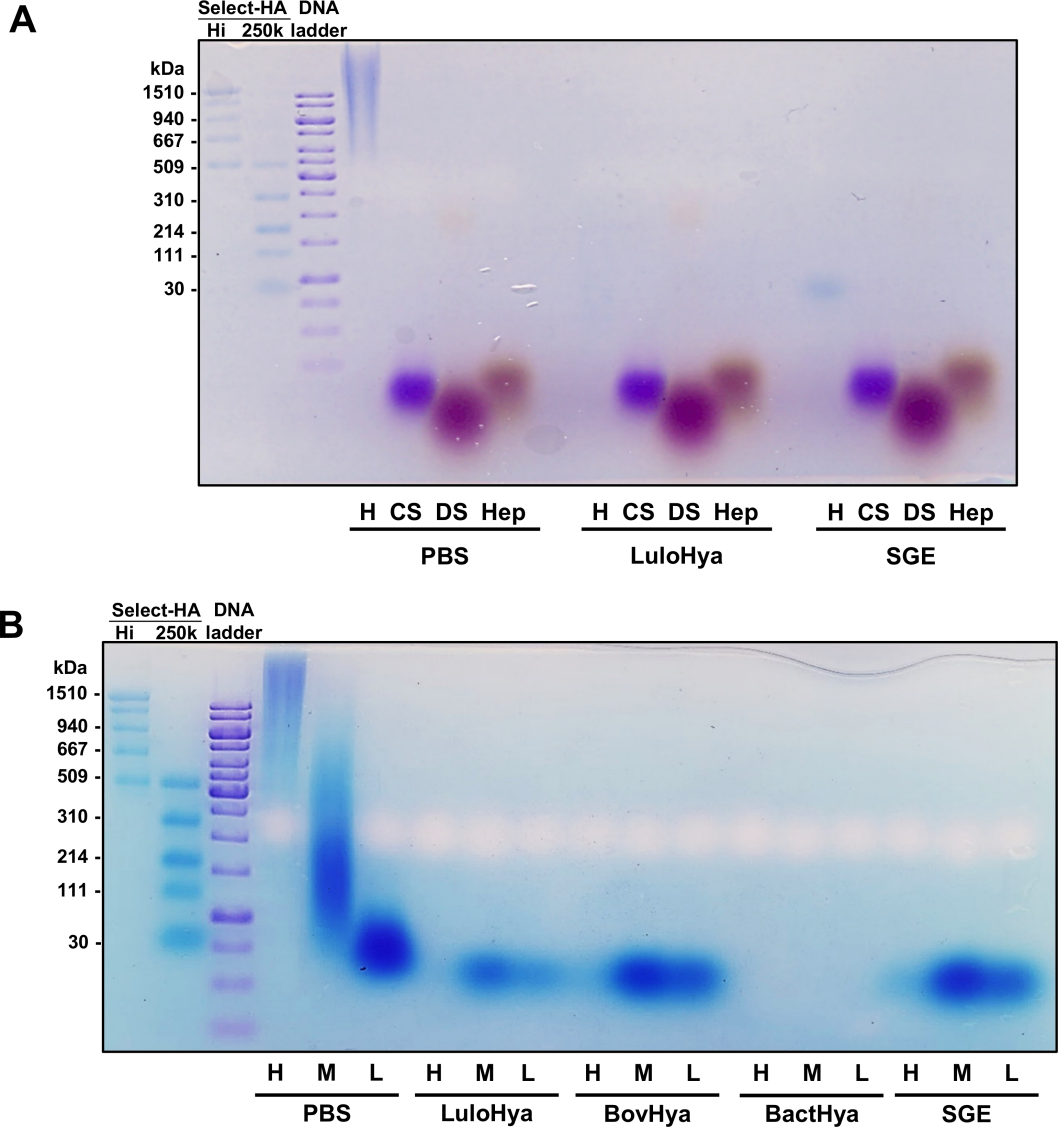
Specificity of hyaluronidase activity of LuloHya. **(A)** Five micrograms of high molecular weight HA (H), chondroitin sulfate B (CS), dextran sulfate (DS) and heparin (Hep) were fractionated on a 1.2% agarose gel alone or after incubation with LuloHya or *Lu*. *longipalpis* SGE. As molecular weight markers, Select-HA HiLadder, Select-HA 250k (Hyalose) and GeneRuler 1kb DNA ladder were used (Thermo Scientific). **(B)** Five micrograms of high, medium and low molecular weight HA (H, M and L, respectively) were separated on a 1.2% agarose gel alone of after incubation with LuloHya, bovine hyaluronidase (BovHya), *Streptomyces hyalurolyticus* hyaluronidase (BactHya) and *Lu*. *longipalpis* SGE.

To investigate pH and ionic strength dependency of LuloHya, a series of enzymatic digestion of HA reactions at different pH and ionic strength conditions were carried out. Recombinant LuloHya shows higher hyaluronidase activity at acidic pH, peaking at pH 6 where no traces of HA were detected (Fig 3E). Therefore, LuloHya belongs to the neutral (pH 5–8) active class of hyaluronidases [13]. Hyaluronidase activity is also dependent on ionic strength, being most active at lower NaCl concentration (25–100 mM, Fig 3F). Because some hyaluronidases can digest other types of substrates, the substrate specificity of LuloHya was determined using different glycosaminoglycans. Both SGE and LuloHya specifically cleave HA but failed to digest other tested components of the extracellular matrix such as chondroitin sulfate B, dextran sulfate or heparin (Fig 4A), confirming that LuloHya is specific for HA processing. Like bovine hyaluronidase, LuloHya and SGE effectively break HA of high, medium and low MW down to 27 kDa. In contrast, hyaluronidase isolated from the bacteria *Streptomyces hyalurolyticus* performs a complete cleavage of glycosaminoglycans (Fig 4B).

### LuloHya and Lundep as the salivary spreading factors in *Lu*. *longipalpis*

**Fig 5:**
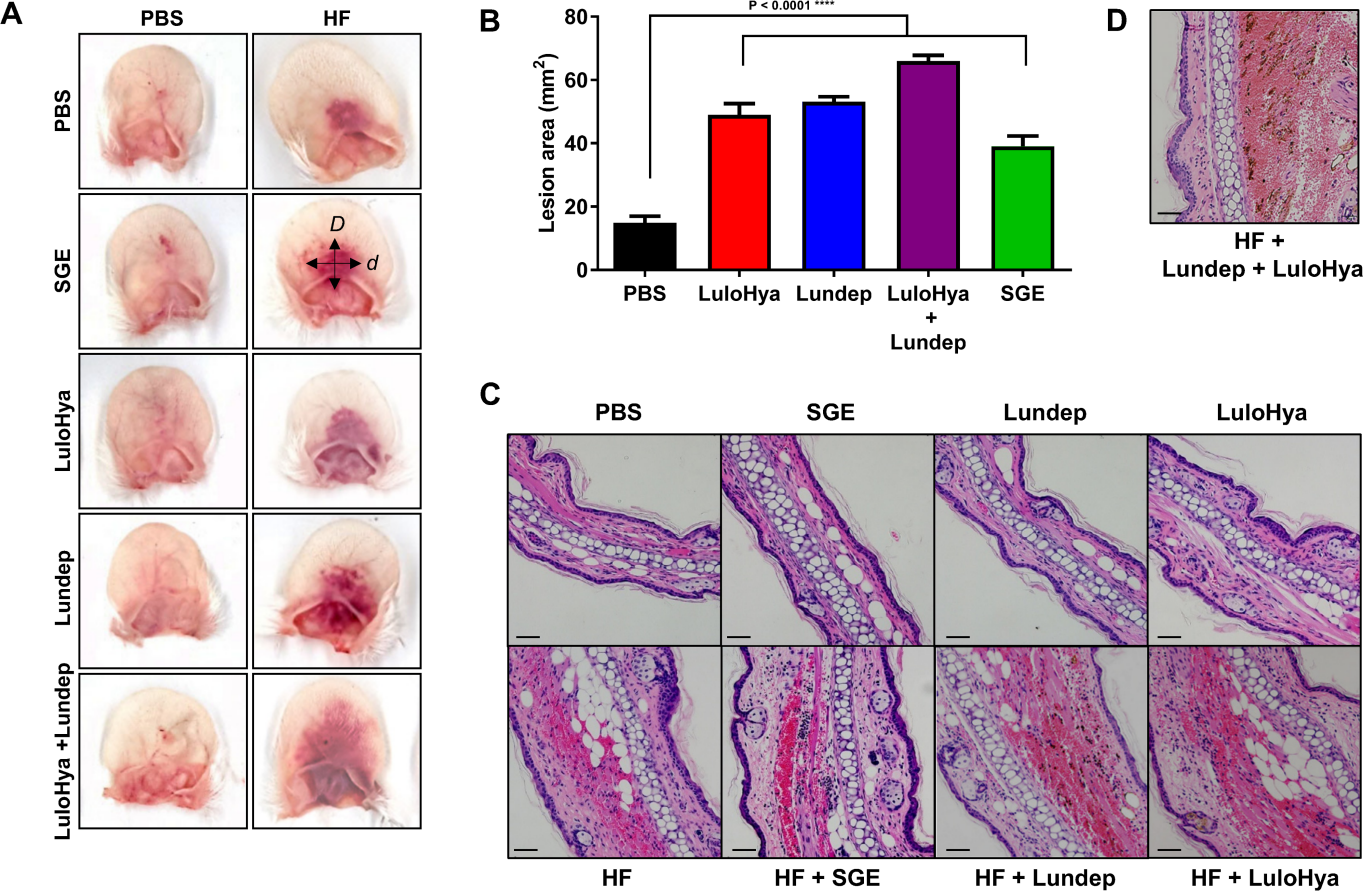
LuloHya and Lundep enhance dermonecrotic lesions caused by HF injection. **(A)** Ears of animals injected intradermally with 3 μg of HF along with SGE (equivalent to 2 SG), 10 μg of LuloHya, 10 μg of Lundep or a combination of 5 μg of LuloHya and Lundep (HF column) showed larger lesions than ears injected with HF and PBS. Left column pictures (PBS) show mice ears injected with SGE, LuloHya, Lundep and the combination of both proteins in the absence of HF. Two hours after injection, ears were excised and measurements of the hemorrhage area were taken. **(B)** Lesion area in all groups injected along HF was significantly greater than control (mice ears injected with PBS and HF). The lesion area (mm) was calculated as follows, Area = $\frac{D*d*\pi }{4}$ where *D* is the longest and *d* the smallest lesion diameter. Multiple comparisons were done by one-way ANOVA (****: p<0.0001). Bars indicate SEM. **(C)** Histological sections of mice ears inoculated with HF were characterized by moderate expansion of the sub-epithelial tissues by a mixture of edema fluid and infiltrating leukocytes. Ear sections inoculated with HF and SGE, LuloHya or Lundep showed acute hemorrhage and inflammation. Mice ears from control groups (without HF) were within normal histological limits. **(D)** Histological ear sections from mice inoculated with HF and both recombinant proteins LuloHya and Lundep showed greater areas of hemorrhage and edema than the inoculation of HF with the recombinant proteins separately. Bar scales indicate 50 μm.

To investigate the biological activity of LuloHya and Lundep and their possible implication as salivary spreading factors, a dermonecrotic assay was performed. Whole ears of naïve mice inoculated with 3 μg of hemorrhagic factor (HF) alone or in combination with SGE (2 pairs), LPS-free LuloHya (10 μg) and LPS-free Lundep (10 μg) were excised after 2 h and the macroscopic changes in the skin of treated animals were measured. All samples were submitted for histological processing and evaluation. The macroscopic hematoma recorded in animals treated with HF in combination with Lundep, LuloHya and SGE groups resulted in a significantly larger area (2.6–4.2-fold increase) when compared to the control group (PBS+HF, Fig 5A and 5B, P<0.0001). Interestingly, combining both recombinant proteins (5 μg each) with HF resulted in a significantly larger area of erythema when compared with that observed when both proteins were injected separately (Fig 5A and 5B).

Histologically, sections of ears collected 2 h post-exposure to HF alone were characterized by moderate expansion of the sub-epithelial tissues by a mixture of edema fluid and infiltrating leukocytes (most often neutrophils). The blood vessels within the affected regions were often dilated and congested and, in some areas, red blood cells were present free within the interstitial tissues immediately surrounding the congested vasculature (hemorrhage). Ear sections from animals injected with HF in combination with Lundep contained focal and extensive areas of acute hemorrhage and inflammation most visible within the layer of the sub-epithelial stroma containing skeletal muscle. Inflammatory cells (neutrophils) were also present in the superficial dermis, between many of the adnexal structures. For the HF+LuloHya group, the changes in these tissues were similar to those described in the HF+Lundep group; however, the superficial dermis was more noticeably expanded by edema fluid and contained a more prominent and predominantly neutrophilic inflammatory cell infiltrate. Sections of ears from mice injected with HF in combination with SGE exhibited milder pathological changes that were characterized by mild sub-epithelial hemorrhage (also associated with skeletal muscle layer) but a much less prominent inflammatory infiltrate and minimal edema. The ears from animals in the control group (without HF) were found to be within normal histological limits (Fig 5C). Ear sections from mice injected with HF and both LuloHya and Lundep showed the most significant areas of stromal hemorrhage and edema. These ear sections were markedly expanded by hemorrhage that ranged from focally extensive to larger coalescing regions and, in some cases, completely obscured the normal dermal structures of the ear (collagen, pilosebaceous units, and skeletal muscle fibers). Inflammation within the superficial dermis was predominantly neutrophilic and of moderate abundance (Fig 5D). Taken together, these results demonstrate that LuloHya and Lundep can enhance the local effect of HF and can potentially act as the spreading factor in the saliva of *Lu*. *longipalpis*, helping in the dissemination of other salivary molecules.

### LuloHya and Lundep can promote neutrophil recruitment

**Fig 6:**
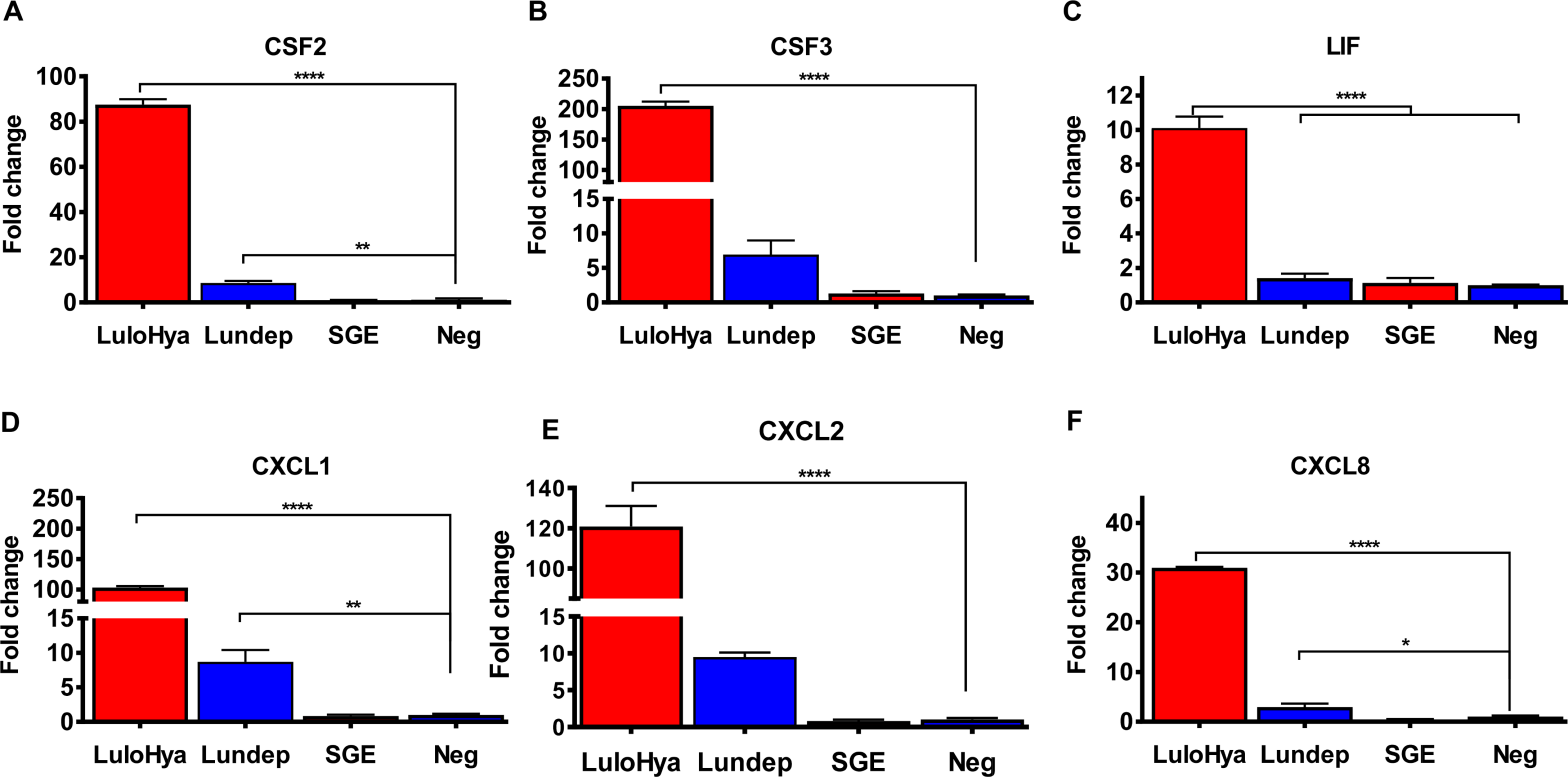
Validation of gene expression of cytokines and chemokines from HMVEC cells in the presence of LuloHya, Lundep or SGE. RT-PCR for validation of gene expression results obtained with the Human Cytokines & Chemokines RT^2^ Profiler PCR Array PAHS-150ZD. Specific set of primers were used to amplify **(A)** CSF2; **(B)** CSF3; **(C)** LIF; **(D)** CXCL1; **(E)** CXCL2 and **(F)** CXCL8. Biological triplicates and technical duplicates were analyzed. Negative controls consisted of cDNA isolated from HMVEC cells incubated with incomplete medium. Results are expressed as the fold change of the gene expression normalized against the standard gene HPRT1 (NM_000194). Multiple comparisons were done by one-way ANOVA (****: p<0.0001; **: p<0.01; *: p<0.05). Bars indicate the SEM.

**Fig 7:**
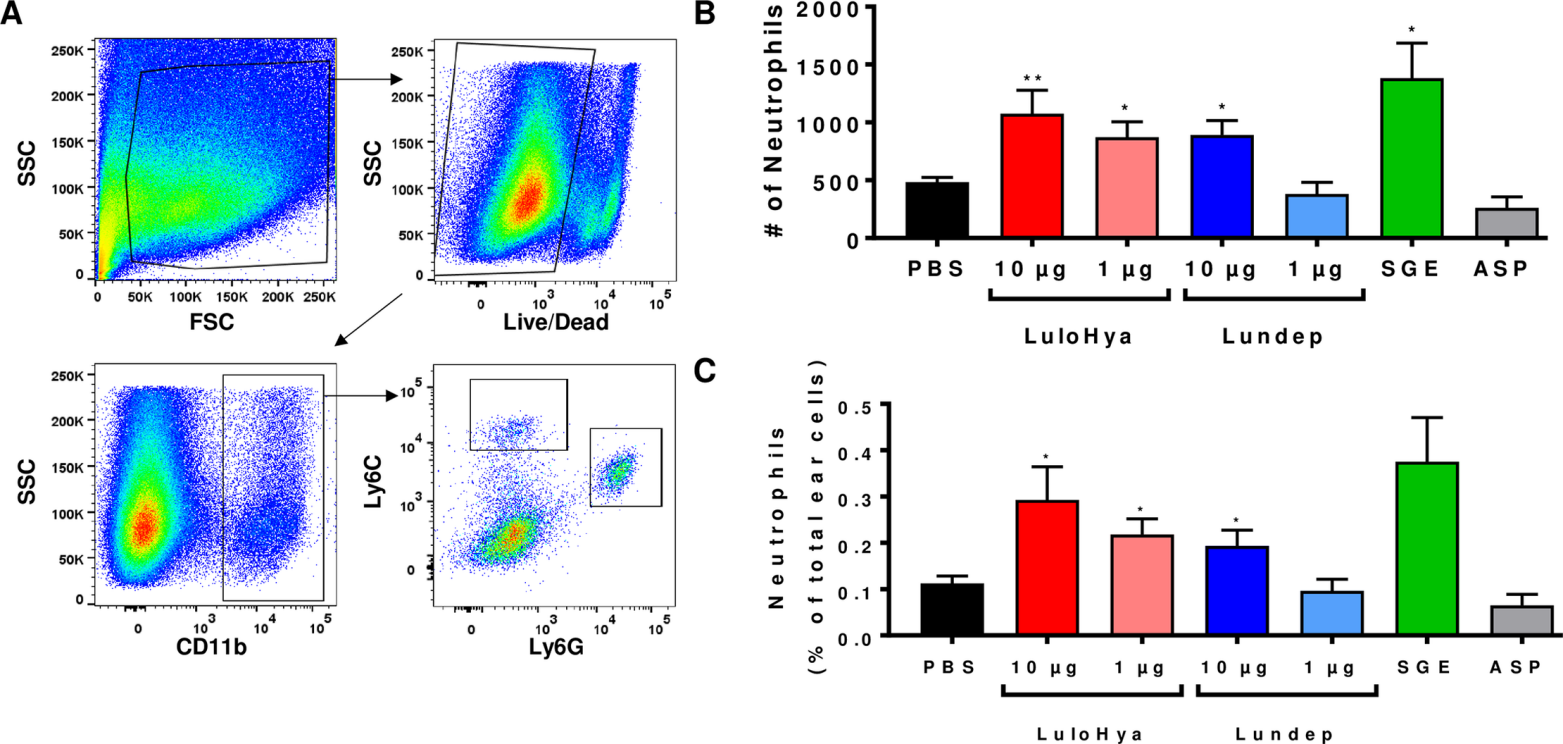
*In vivo* recruitment of polymorphonuclear leukocytes by LuloHya, Lundep or SGE in mice. Recruitment of neutrophils to the inoculation site was determined by flow cytometry analysis. (**A**) Gating strategy analysis based on the CD11b expression to identify neutrophils (Ly6G^+^Ly6C^int^) and inflammatory monocytes (Ly6G^-^Ly6C^hi^). Mice (C57BL/6) were intradermally inoculated with LuloHya (10 μg and 1 μg), Lundep (10 μg and 1 μg), *Lu*. *longipalpis* SGE (equivalent to 2 SG pairs) and PBS alone as a negative control. Ears injected with 1 μg of non-related salivary protein from *Ae*. *aegypti* (ASP) were also included. After 2 h, the ears were collected and processed for flow cytometry analysis. **(B)** Total count of neutrophils per ear. (**C**) Frequency of neutrophils in mouse ears. Results (of at least 2 independent experiments) are shown as mean +/- SEM. Comparisons with PBS group were done by Mann-Whitney U test (**: p<0.01; *: p<0.05).

Neutrophils are the first leukocytes to be recruited to an inflammatory site such as the one caused by blood feeding arthropods and represent the first line of defense against pathogen infections [25, 26]. To examine the role of LuloHya and Lundep in neutrophil recruitment, human dermal microvascular endothelial cells (HMVEC) were treated with 1 μM of LuloHya, Lundep (LPS-free) and SGE (10 pairs) or culture media alone and the gene expression of 84 cytokines and chemokines were measured using a qPCR array. After 4 h of incubation, genes coding for CCL2, CCL20, CSF2, CSF3, CX3CL1, CXCL1, CXCL10, CXCL2, CXCL8, LIF, LTB and TNF were upregulated in the presence of LuloHya, whereas IL-11 and PPBP were downregulated. When cells were treated with Lundep, genes coding for CSF2, CSF3, CXCL1 and CXCL2 were also overexpressed. Additionally, IL-1 alpha, IL-5, SPP1, IFN gamma and chemokines PPBP, CCL3 and CXCL13 were downregulated in the presence of Lundep (Table 1). These results (upregulated genes with more than 10-fold change) were validated by qPCR in a separate experiment (Fig 6). The lack of effect of SGE on cytokine or chemokine gene expression change might be a result of low individual salivary protein concentration compared to the amount of recombinant protein tested. Because CXCL8 was upregulated upon stimulation *in vitro*, we also investigated the ability of these molecules to induce neutrophil infiltration *in vivo*. For this experiment, the ears of C57BL/6 mice were intradermally injected with either LPS-free LuloHya (10 μg and 1 μg), Lundep (10 μg and 1 μg), or *Lu*. *longipalpis* SGE (equivalent to 2 pairs). As a negative control ears were injected with PBS alone or with a non-related salivary protein from the mosquito *Aedes aegypti* which was expressed and purified in the same manner to rule out unspecific cell recruitment. After 2 h, the ears were collected and polymorphonuclear leukocyte infiltration was analyzed by flow cytometry. Myeloid live cells were gated based on the CD11b expression and then further characterized as Ly6G^+^Ly6C^int^ neutrophils or Ly6G^-^Ly6C^hi^ inflammatory monocytes (Fig 7A). We observed a significant increase in the total number and frequency of neutrophils in the ears of mice injected with LuloHya when compared with PBS-injected mice. Salivary gland extracts from female *Lu*. *longipalpis* also increased infiltration of neutrophils in mice while Lundep alone slightly increased cell recruitment (Fig 7B and 7C). The neutrophil recruitment appears to be dose dependent as higher doses promoted greater cell recruitment at the inoculation site. This effect is specific for the tested sand fly salivary proteins, as the *Ae*. *aegypti* protein did not alter the cell recruitment. Taken together, our results demonstrated that LuloHya and SGE actively cause neutrophil infiltration to the mouse ears and may serve as a vehicle for establishment of *L*. *major* infection. The relevance of cell recruitment to the bite site, for blood feeding, remains to be elucidated.

**Table 1:** Cytokine and chemokine gene expression in the presence of salivary proteins LuloHya and Lundep.

Gene symbol	Refeq Accession	Description	LuloHya (fold change)	Lundep (fold change)
CCL2	NM_002982	Chemokine (C-C motif) ligand 2	7.2183	*
CCL20	NM_004591	Chemokine (C-C motif) ligand 20	7.7902	*
CSF2	NM_000758	Colony stimulating factor 2 (granulocyte-macrophage)	64.5197	6.0765
CSF3	NM_000759	Colony stimulating factor 3 (granulocyte)	286.3565	13.5783
CX3CL1	NM_002996	Chemokine (C-X3-C motif) ligand 1	4.0325	*
CXCL1	NM_001511	Chemokine (C-X-C motif) ligand 1 (melanoma growth stimulating activity, alpha)	64.0741	8.4166
CXCL10	NM_001565	Chemokine (C-X-C motif) ligand 10	4.5054	*
CXCL2	NM_002089	Chemokine (C-X-C motif) ligand 2	89.9885	9.2742
CXCL8	NM_000584	Interleukin 8	22.4971	*
LIF	NM_002309	Leukemia inhibitory factor (cholinergic differentiation factor)	10.8654	*
LTB	NM_002341	Lymphotoxin beta (TNF superfamily, member 3)	7.37	*
TNF	NM_000594	Tumor necrosis factor	7.3191	*
IL11	NM_000641	Interleukin 11	-4.4025	*
PPBP	NM_002704	Pro-platelet basic protein (chemokine (C-X-C motif) ligand 7)	-4.5895	-5.4519
CCL3	NM_002983	Chemokine (C-C motif) ligand 3	*	-4.1895
CXCL13	NM_006419	Chemokine (C-X-C motif) ligand 13	*	-5.9248
IFNG	NM_000619	Interferon, gamma	*	-4.6485
IL1A	NM_000575	Interleukin 1, alpha	*	-4.7792
IL5	NM_000879	Interleukin 5 (colony-stimulating factor, eosinophil)	*	-5.3029
SPP1	NM_000582	Secreted phosphoprotein 1	*	-4.1318

*Fold change values were either not greater than 4 or less than -4.

### Mice immunized against LuloHya and Lundep control *L*. *major* infection

**Fig 8:**
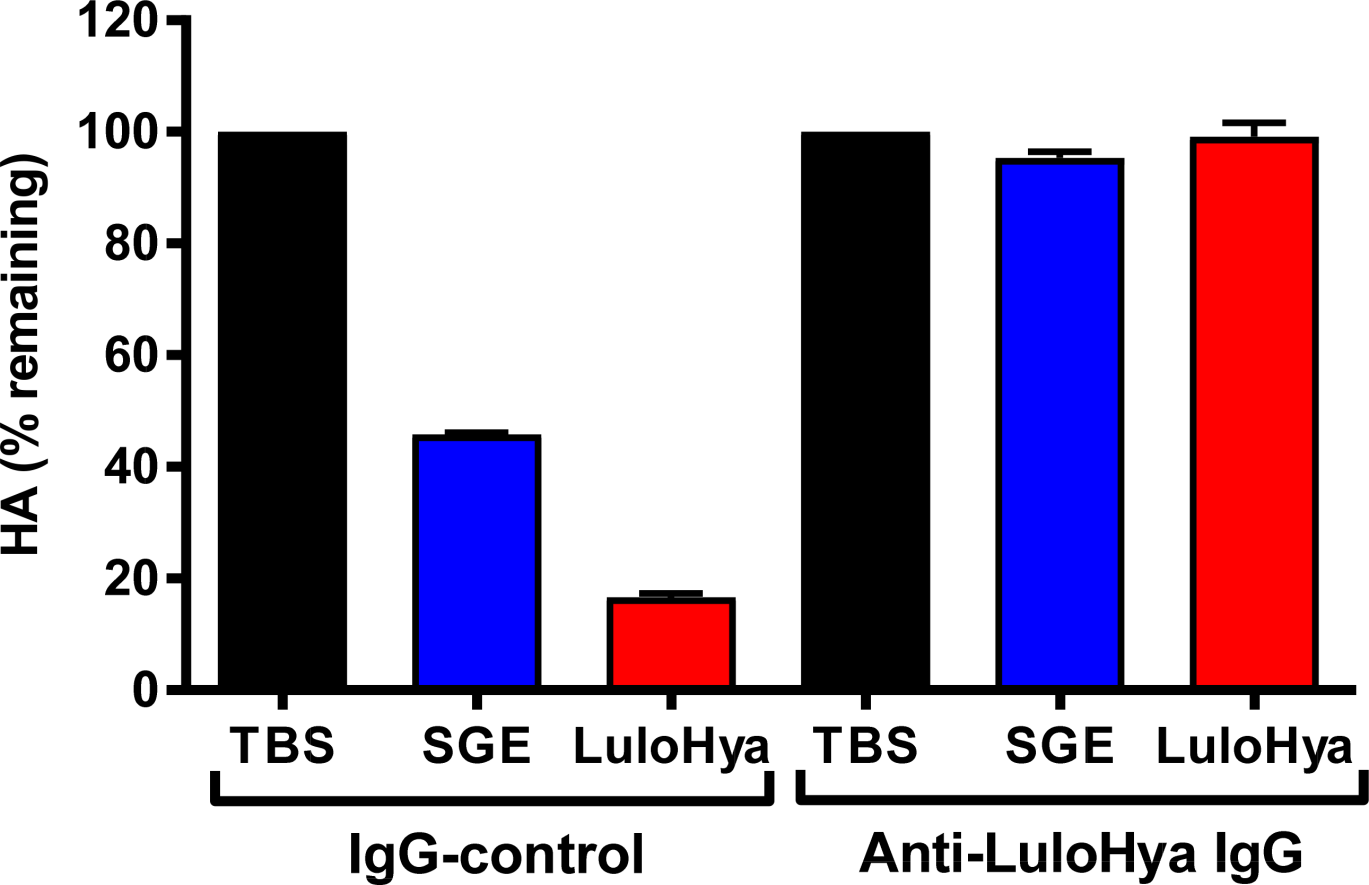
Anti-LuloHya antibodies block the hyaluronidase activity of SGE and LuloHya. Hyaluronidase activity of *Lu*. *longipalpis* SGE and 10 nM LuloHya in the presence of 5 μg of rabbit IgG-control (pre-immune IgG) or 5 μg of rabbit anti-LuloHya IgG. As negative controls, TBS was added instead of SGE or recombinant protein. Biological triplicates were tested. Bars indicate SEM.

**Fig 9:**
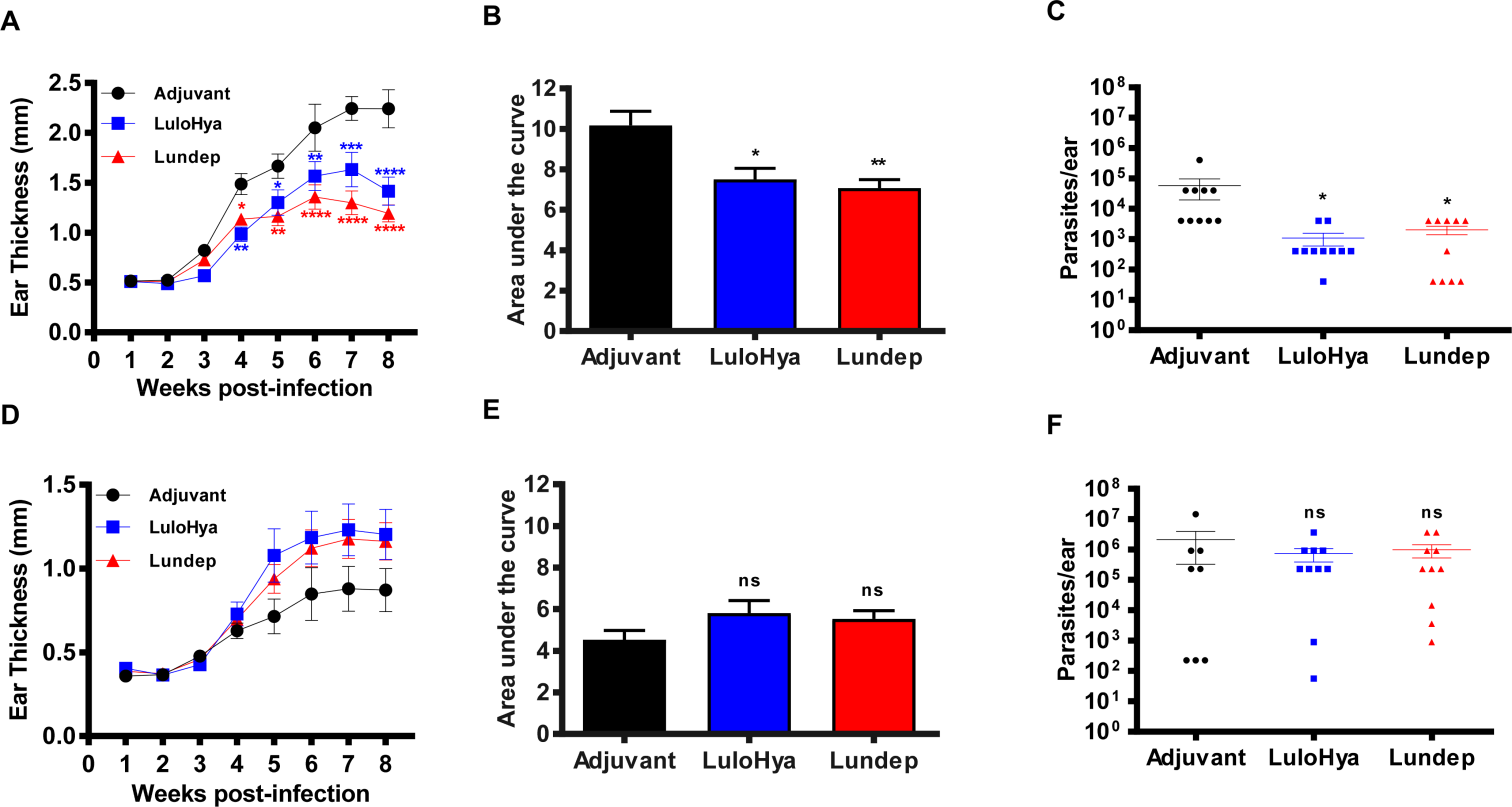
Vaccination studies with LuloHya and Lundep against *L*. *major* infection. **(A)** Lesion size of C57BL/6 mice due to *L*. *major* infection steadily increased from the second week of the follow-up period until it stabilized or started to decrease after week 7 in all animals. For control animals (only immunized with Magic Mouse Adjuvant) lesion size was higher than vaccinated groups from week 4 onwards. The symbols represent the lesion size mean of 10 ears ± SEM analyzed by analysis of variance. **(B)** Lesion size values of C57BL/6 mice were converted to the area under the curve (AUC) showing that mice immunized against either LuloHya and Lundep presented significantly reduced lesions. **(C)** Parasite load of ears from C57BL/6 mice vaccinated with LuloHya or Lundep was significantly lower than control group (P<0.05). **(D)** *L*. *major* lesion size in B-cell-deficient B6.129S2-*Ighm*^*tm1Cgn*^/J mice. **(E)** There are no statistical significant differences in lesion size of B6.129S2-*Ighm*^*tm1Cgn*^/J mice immunized with either LuloHya, Lundep or adjuvant. **(F)** Parasite load of ears from B6.129S2-*Ighm*^*tm1Cgn*^/J mice vaccinated with LuloHya or Lundep showed no differences with the control group. Multiple comparisons were done by one-way ANOVA (****: p<0.0001; ***: p<0.001; **: p<0.01; *: p<0.05; ns: non-significant). Bars indicate SEM.

Chagas *et al* [9] found that antibodies against Lundep block the DNase activity of female SGE and proposed the potential use of Lundep as an anti-*Leishmania* vaccine. In this work, we also found that anti-LuloHya antibodies can effectively block the hyaluronidase activity of LuloHya and SGE *in vitro* (Fig 8). Based on these results we carried out a vaccination experiment to test whether antibodies against LuloHya and Lundep can neutralize the endonuclease and hyaluronidase activity of *Lu*. *longipalpis* saliva *in vivo* thus protecting against cutaneous leishmaniasis in mice. Animals (C57BL/6 mice) were immunized with LuloHya and Lundep and challenged via needle injection with 10^3^ *L*. *major* metacyclic promastigotes mixed with SGE (equivalent to 1 pair) and the ear lesion size was measured weekly for 8 weeks. Mice immunized against LuloHya and Lundep showed a significant reduction in the lesion size (2-fold smaller) caused by *L*. *major* infection than those recorded in mice treated with adjuvant alone (P<0.05 and P<0.01, respectively, Fig 9A and 9B). Furthermore, vaccinated mice with either LuloHya or Lundep also showed a markedly reduced parasite load (28–53 fold) in their ears (P<0.05, Fig 9C) than control mice. To determine whether this protection against *L*. *major* infection is due to cellular or humoral immune response to LuloHya and Lundep, we carried out a similar vaccination experiment in B-cell-deficient (B6.129S2-*Ighm*^*tm1Cgn*^/J) mice. Immunization, parasite challenge and lesion measurement were carried out exactly as described above. Results shown in Fig 9D and 9E, demonstrated that the protective effect of LuloHya and Lundep against *L*. *major* infection is antibody-mediated response since the protection against parasite infection was abolished in B-cell-deficient mice. Parasite load showed no significant differences among the 3 groups (Fig 9F).

### Antibodies against LuloHya and Lundep do not cross react with *L*. *major* parasites

Mouse anti-LuloHya and anti-Lundep antibodies did not recognize *L*. *major* extract when analyzed by ELISA. Ninety-six well plates were coated with either LuloHya, Lundep or *L*. *major* extract. Anti-LuloHya antibodies strongly reacted with LuloHya but not Lundep or *L*. *major* extract. Likewise, anti-Lundep antibodies only recognized Lundep. A strong positive reaction was observed between anti-tubulin (positive control) and *L*. *major* due to the presence of tubulin proteins in the parasite membrane, ensuring parasite protein antigenicity (S1A Fig). Comparable results were obtained by immunofluorescence microscopy, where neither mouse anti-LuloHya nor anti-Lundep antibodies recognized *L*. *major* parasites (S1B Fig).

### LuloHya plays a key role in blood feeding

Because rabbit IgG blocked the hyaluronidase activity of LuloHya and SGE *in vitro* (Fig 8), we also investigated the role of LuloHya in *Lu*. *longipalpis* blood feeding in mice. Blood feeding success was tested on passively immunized mice with anti-LuloHya or pre-immune IgG as a control. Among the sand flies that attempted to feed on mice with circulating anti-LuloHya antibodies only 22% succeed in ingesting blood compared to 51% success in the control group (reduction of 56.86%, P<0.0001, *X*^*2*^ test, S2A Fig). In our experiments, a 12 min period was chosen as the appropriate time to obtain around 60% of blood feeding success in the control group (sand flies fed on mice with circulating pre-immune IgG). This effect was maintained when sand flies were blood fed for a second time on mice passively immunized (28% of engorged sand flies fed on mice with anti-LuloHya antibodies versus 48% of engorged sand flies fed on control mice, P = 0.0009, *X*^*2*^ test). When sand flies are allowed to feed for a longer period of time (45 min), the trend is maintained; there is still a lower percentage of sand flies that successfully acquired blood when feeding on mice with anti-LuloHya antibodies (81%) when compared with the sand flies fed on control mice (90.2%), however, the difference was not statistically significant. The relevance of the LuloHya and Lundep on other physiological parameters such as blood meal size or oviposition was also assayed. We compared blood meal size of fully engorged sand flies fed on animals passively immunized against LuloHya and Lundep [9]. No significant difference in blood meal size and oviposition rate of sand flies blood fed on immunized mice compared to sand flies fed on control mice were observed (S2B and S2C Fig, [9]).

## Discussion

Sand fly saliva contains a vast array of pharmacological potent substances, including endonucleases and hyaluronidases, that counteracts vertebrate hemostasis and enhance pathogen transmission. Commonly found in poisonous animals, hyaluronidases and non-specific endonucleases are thought to be essential for the spreading of toxins and other venom component by compromising the integrity of extracellular matrix and significantly increasing the diffusion rates of other molecules [27, 28]. Similarly, both enzymes (EST or enzymatic activity) are found in salivary glands of blood feeding arthropods [9, 15, 17, 20, 29–31]. Notably, these two enzymes appear to be mostly expressed in salivary glands as a pair, where their combination may help the diffusion of other salivary components and assisting blood meal intake by lowering the local viscosity caused by the release of host DNA and other extracellular matrix (ECM) components, and as an anticoagulant by inhibiting the intrinsic pathway of coagulation. Sand fly mouthparts penetrate no more than 0.5 mm of the host skin and thus can only reach the superficial capillaries. These are most of the time closed, as skin capillaries serve a function on heat dissipation in addition to skin nutrition. The capillary flow is regulated by the pre-capillary arteriolar sphincter. The vasodilators in sand fly saliva have to travel through the skin tissues deeper into the arteriolar bed, and this task may be facilitated by salivary spreading factors [32]. Although hyaluronidase activity was known to be present in sand fly saliva for several years [15, 16, 19, 21, 33], their recombinant salivary proteins have never been produced. In this work, we successfully expressed LuloHya, the recombinant hyaluronidase from *Lu*. *longipalpis* salivary glands to further characterize it and its relevance in blood feeding and parasite transmission.

Gene expression and enzymatic activity of LuloHya was exclusively found in adult female sand flies. These two findings suggest that LuloHya is involved in blood feeding. Moreover, its sequence contains a signal peptide and this protein, along with other salivary proteins, is secreted during blood feeding, as its activity is highly reduced afterwards [19]. LuloHya is catalogued as an endo-N-acetyl-hexosaminidase, an enzyme class that includes mammalian and hymenopteran hyaluronidases [14, 16, 20]. LuloHya specifically cleaved HA and did not break down other components of the extracellular matrix tested in our study. Lack of hydrolysis of chondroitin sulfate B was already ascribed for LuloHya but a moderate chondroitinase activity (due to the breakdown of chondroitin sulfate A and C) had been observed [20]. LuloHya hyaluronidase activity is dependent on pH, showing its highest activity at acidic pH, being concordant with studies in other insect hyaluronidases [15, 18, 20]. There is a pH gradient through the epidermis, varying from acidic values on the skin surface (pH 4–6) to physiological pH of 7.4 in inner skin layers [34]. Interestingly, LuloHya would remain active through this pH range.

Hyaluronidases and endonucleases appear to come in a two-pack in insect saliva. Either both transcripts are transcribed resulting in functional proteins or none of them are. For instance, in the saliva of *Ae*. *aegypti* or *Anopheles* spp. mosquitoes neither one of these transcripts are present or active [16, 35, 36] whereas *Culex quinquefasciatus* saliva shows a potent hyaluronidase and endonuclease activity [16, 29]. *Culex* mosquitoes preferentially feed on birds [37]; therefore, both activities seem essential for counteracting the high density of avian blood meal, due to the presence of nucleated blood cells. In the case of sand flies, they feed on birds as well as mammals; being the host preference dependent on the sand fly species and the host availability [38–40]. Sand flies, as hematophagous insects, require a blood meal for egg development. Their blood feeding strategies consist of lacerating tissues and feeding from skin hemorrhages. Tissue damage caused by lacerating the host’s skin can release the vertebrate cell contents at the bite site, including nucleic acids and HA among other ECM components. Local release of these components can increase the local viscosity at the bite site. Hyaluronidase and endonuclease activities have been mainly found in the saliva of insects that use pool feeding strategies as well as in poisonous snakes and bee venom where it has been proposed to potentiate the biological effect of other toxins present in the venom. Venom hyaluronidases can contribute to systemic envenomation by accelerating venom absorption and diffusion [41]. Based on these finding, anti-hyaluronidases antibodies and inhibitors have been considered as potential treatments to attenuate local and systemic effect of snake poisoning [42]. Interestingly, antibodies against LuloHya completely abrogate the enzymatic activity of recombinant LuloHya and SGE. Our results also demonstrate the role of LuloHya in blood feeding, since passively immunized mice with anti-LuloHya antibodies significantly reduced the feeding success of *Lu*. *longipalpis*. Similar results were obtained when feeding sand flies on mice with circulating anti-Lundep antibodies [9]. Accordingly, blocking the salivary hyaluronidase and endonuclease activities of *Lu*. *longipalpis* can result in a more viscous skin environment, preventing the dispersion of other salivary components involved in blood feeding as well as making it more difficult to ingest a blood meal. Sand fly blood feeding was impaired when salivary hyaluronidase was blocked *in vivo*, as shown in our blood feeding success assays on passively immunized mice with anti-LuloHya antibodies. In these experiments, sand flies were allowed to feed for a short period of time (12 min). Interestingly, no differences were found on the feeding success rate using naïve sand flies *versus* sand flies that had already fed on passively immunized animals, ruling out the possibility of selection of a resistant population after vaccination with these salivary proteins. If they are permitted to feed for a longer period (45 min) the trend is maintained: there is still a lower percentage of sand flies that successfully acquired blood when feeding on mice with anti-LuloHya antibodies, however, the difference is not statistically significant. It should be noted that feeding success experiments in the laboratory were carried out on anesthetized animals. These differences in required time to successfully acquiring a blood meal may be of epidemiological relevance in nature as longer feeding time can lead to awareness of the host and trigger defensive behavior of the host preventing a completion of a blood meal or even causing the death of the insect. Although sand flies take longer to blood feed in a hyaluronidase-blocked environment, they eventually ingest a similar amount of blood and no differences in other biological parameters such as oviposition rates were noticed.

Bacterial hyaluronidases cleave HA down to disaccharides, thus helping bacterial nutrition [43]. However, LuloHya breaks HA down to fragments of 27 kDa, in a similar fashion as bovine hyaluronidase digestion of HA. During a skin injury, degradation of HA into small polymer fragments can lead to the activation of an alarm signal that triggers an immune response. Immunology of HA is a very complex field as different molecular sizes of HA produce contrary effects. While high molecular weight HA causes immune suppression small HA metabolites have been incriminated in the inflammatory stage of a healing wound [44]. Increased concentration of HA fragments in injured tissues, like the one caused by sand fly bites, inhibit fibrocyte differentiation and collagen deposition to allow macrophages to move freely around the injured site to phagocyte debris and clear any possible infectious agents [45]. Interestingly, our study shows that expression of neutrophil-specific CXC chemokines and colony stimulating factors were upregulated in HMVEC cells after incubation with LuloHya, and Lundep. Neutrophil-specific CXC subfamily of chemokines has been shown to be involved in leukocyte chemotaxis and inflammatory responses [46]. Granulocyte-colony stimulating factors (CSF2 and CSF3) and leukemia inhibitory factor (LIF) are cytokines involved in leukocyte activation and differentiation. The effect of these 2 salivary molecules on cytokine and chemokine production could be attributed to either the salivary enzymes or their metabolites. The latest hypothesis agrees with other authors findings who have shown that small HA fragments induce leukocyte chemotaxis and expression of inflammatory cytokines [44, 47–49]. Moreover, HA cleavage by hyaluronidase opens tissue spaces that facilitate neutrophil invasion. These results were consistent with the acute hemorrhage, edema and inflammation involving a massive infiltration of leukocytes, especially neutrophils, observed in the dermonecrotic assay in mice. Moreover, neutrophil infiltration in the inoculation site was demonstrated *in vivo* by flow cytometry experiments in the presence of these two proteins and SGE.

We hypothesize that in the *Lu*. *longipalpis*—*L*. *major* transmission model, both proteins cooperate at the bite site promoting a favorable environment for *Leishmania* parasite: LuloHya would enhance neutrophil recruitment whereas Lundep would protect *L*. *major* from the leishmanicidal activity of neutrophil extracellular traps [9]. Ultimately, *L*. *major* survival in neutrophils would be enhanced and used as Trojan horses for further invasion of macrophages [50]. Previous studies on Lundep and bovine hyaluronidase treated these two salivary proteins as virulence factors, since *L*. *major* lesions were enlarged when these proteins were co-administered with the parasite [9, 16]. In our work, we showed that mice were able to control *L*. *major* infection when vaccinated against either Lundep or LuloHya. Moreover, when using B-cell-deficient mice, the protection seen in C57BL/6 mice was abrogated, indicating that it is due to a humoral-mediated immune response. Antibodies against LuloHya and Lundep did not recognize *L*. *major* parasites either by immunofluorescence or ELISA. Therefore, the possibility of a direct effect of anti-LuloHya or anti-Lundep on the parasite was ruled out. The protective effect of sand fly saliva or specific salivary proteins has been generally attributed to a cellular immune response at the bite site (delayed-type hypersensitivity) [51]. Although the protecting effect of neutralizing antibodies against salivary proteins has been neglected [4, 52], we have shown here that neutralizing antibodies against LuloHya and Lundep play a role on the *Leishmania* infection development, as determined by the ear lesion and parasite load measurements.

Although the sand fly–parasite combination used in this study does not occur in nature, *Lu*. *longipalpis* is a permissive sand fly that supports *L*. *major* infection in laboratory conditions. Furthermore, our laboratory has a well-established murine model of infection for this pair and it allowed us to compare our results with previous publications [9, 53, 54]. We believe that results showed in this article could be extrapolated to the natural setting where *Phlebotomus papatasi* acts as the natural vector of *L*. *major*. *Phlebotomus papatasi* has both enzymatic activities in their salivary glands; hyaluronidase [20] and endonuclease ([55], S3 Fig).

In conclusion, we provide new insights of LuloHya and Lundep, the proteins responsible for the hyaluronidase and endonuclease activities present in the salivary glands of female *Lu*. *longipalpis*. These two proteins are crucial for accelerating the blood feeding process by reducing local viscosity at the bite site and disseminating other salivary components. In our model of study, LuloHya and Lundep promote neutrophil recruitment to the bite site that may serve as a vehicle for establishment of *L*. *major* infection. Our vaccine results clearly demonstrate the protective effect of antibodies against LuloHya and Lundep indicating that these two salivary proteins are promising vaccine candidates against *Leishmania* infection. Furthermore, it has been suggested that a saliva-based vaccine could be theoretically be cross-protective between phylogenetically related vector species [4]. Therefore, the potential use of these *Lu*. *longipalpis* proteins to protect infection of different *Leishmania* species transmitted by other sand flies should be considered. These aspects need to be evaluated with the natural sand fly—*Leishmania* parasite combination. The work presented herein is another example of the complexity of the biology of sand fly blood feeding and the potential use of vector salivary proteins as transmission blocking vaccines. Efforts to unravel these complex mechanisms should be encouraged.

## Materials and methods

### Ethics statement

Public Health Service Animal Welfare Assurance #A4149-01 guidelines were followed according to the National Institute of Allergy and Infectious Diseases (NIAID), National Institutes of Health (NIH) Animal Office of Animal Care and Use (OACU). These studies were carried out according to the NIAID-NIH animal study protocol (ASP) approved by the NIH Office of Animal Care and Use Committee (OACUC), with approval IDs ASP-LMVR3 and ASP-LMVR4E.

### Sand fly rearing and salivary gland dissection

*Lutzomyia longipalpis* (Jacobina strain) were maintained using standard procedures in the insectary of the Laboratory of Malaria and Vector Research, NIAID [56]. Sand flies were anesthetized with CO_2_, and SG dissections were manually performed under a stereo microscope in 20 mM phosphate buffer. SGE was prepared by sonication, according to Ribeiro *et al* [57]. For the endonuclease assays, SGE from *Phlebotomus duboscqi* and *P*. *papatasi* from Saudi Arabia and Turkey was used.

### Cloning, expression and purification of LuloHya

LuloHya coding DNA sequence (AF132515) was codon-optimized for mammalian expression and synthesized by BioBasic Inc. VR2001-TOPO vector containing LuloHya sequence (Vical Incorporated) was transformed in One Shot TOP10 Chemically Competent *E*. *coli* (Invitrogen). Plasmid DNA was prepared using the EndoFree plasmid MEGA prep kit (Qiagen, Valencia, CA). Recombinant protein expression was carried out at the SAIC Advance Research Facility (Frederick, MD). Briefly, human embryonic kidney cells (HEK293E; American Type Culture Collection, Manassas, Virginia) were transfected with 1 mg of plasmid DNA and supernatants were collected 72 h after transfection and shipped frozen to our laboratory for protein purification. Recombinant LuloHya was purified by affinity chromatography followed by size-exclusion chromatography, using Nickel-charged HiTrap Chelating HP and Superdex 200 10/300 GL columns, respectively (GE Healthcare Life Science, Piscataway, NJ). All protein purification experiments were carried out using the AKTA purifier system (GE Healthcare Life Science, Piscataway, NJ). Purified protein was separated in a NuPAGE Novex 4–12% Bis-Tris Protein Gels (Life Technologies) and visualized by Coomassie stain. Protein identity was verified by Edman degradation at the Research Technologies Branch, NIAID, NIH.

### Endotoxin removal

Lipopolysaccharides (LPS) can induce an immune response and thus samples containing LPS can generate false results. To ensure the recombinant proteins used in this study were LPS-free LuloHya and Lundep were LPS-decontaminated using the Pierce High-Capacity Endotoxin Removal Resin (Thermo Scientific). Briefly, recombinant protein samples were passed through a column containing 1 ml-bed volume of a resin consisted of cellulose beads covalently attached to a modified polylysine associated with polymyxin B ligands which show high affinity for LPS. Proteins were incubated with the resin for 2 h at room temperature before being eluted with endotoxin-free, sterile PBS. Endotoxin levels were measured by Endosafe nexgen-PTS (Charles River). All samples were below the allowable endotoxin limits stated by the FDA for IV preparations (5 EU/kg).

### Hyaluronidase assay

The hyaluronidase activity was determined by the turbidimetric method as previously described [15] with minor modifications. Briefly, 0.1 mg/ml HA solution was prepared in 25 mM HEPES, 100 mM NaCl, 0.1% BSA, pH 7.3. Four micrograms of HA were incubated with the SGE or 10 nM LuloHya (final concentration). Reaction was run at 37°C for 1 h and stopped by the addition of 100 μl of 10 mg/ml cetylpyridinium chloride, which reacts with HA resulting in turbidity. Plates were read at 540 nm using a VersaMax microplate reader (Molecular Devices). Turbidity corresponds with intact HA. Blanks lacking samples as well as HA were included in all tests.

To characterize the ionic strength dependence of LuloHya activity, HA was prepared in different concentrations of NaCl (25, 50, 100, 250, 500 and 1000 mM). In the case of the pH dependence, hyaluronidase activity was measured using buffers at pH 4, 5 (25 mM acetate), 6 (25 mM MES), 7, 8 (25 mM HEPES), 9 and 12.5 (25 mM CAPS).

The effect of glycosylation of LuloHya on hyaluronidase activity was investigated. The recombinant protein was deglycosylated using the Enzymatic DeglycoMx Kit (QABio), following the manufacturer’s instructions. Both native and deglycosylated recombinant LuloHya were run on a NuPAGE gel and hyaluronidase turbidimetric assay was performed.

### Hyaluronidase activity analysis on agarose gels

Five micrograms of HA of different sizes (high, medium and low molecular weight, which correspond to > 950 kDa, 75–350 kDa and 15–40 kDa, respectively, RD Systems, Minneapolis, MN) were incubated for 1 h at 37°C with SGE (equivalent of 1 SG pair) or LuloHya (10 nM). Other commercial hyaluronidases were included as controls: bovine hyaluronidase (Sigma) and hyaluronidase from *Streptomyces hyalurolyticus* (Sigma), both at 1 Unit per reaction. Samples were run on a 1.2% agarose gel for 30 min at 20 V followed by 2 h 30 min at 40 V. Agarose gels were stained with 0.05% Stains-all (Sigma).

For hyaluronidase activity specificity, other glycosaminoglycan components of the extracellular matrix were also tested, including chondroitin sulfate B, dextran sulfate and heparin (5 μg/sample, Sigma).

### Endonuclease assay

Assays for determine the nuclease activity were carried out as previosuly described [9]. 200 ng of double stranded circular plasmid DNA (VR2001; Vical Inc., San Diego, CA) were incubated with SGE from different *Phlebotomus* species in the presence of 50 mM Tris, 150 mM NaCl, 5mM MgCl_2_, pH 8.0.

### Polyclonal antibody production and blocking activity *in vitro*

Polyclonal antibodies against LuloHya were raised in mice and rabbits. Mice (Balb/c; Charles River, Frederick, Maryland) were IM immunized with 10 μg of LuloHya or PBS as the control group in combination with Magic Mouse Adjuvant (CD Creative Diagnostics, Shirley, NY). Mice were boosted 3 weeks after the first immunization and blood was collected ten days later.

Immunization of rabbits was carried out in Noble Life Science facility (Sykesville, MD) according to their standard protocol (http://www.noblelifesci.com/preclinical-drug-development/polyclonal-antibody-production/). Briefly, animals (female New Zealand white rabbits, 3 month old) were immunized 3-times with 200 μg of recombinant protein (Freund’s complete adjuvant was used for first injection and Freund’s incomplete adjuvant in the last 2 injections) every 21 days and the serum collected at day 72. Rabbit sera (pre-immune and immune) were shipped to our laboratory where purification of IgG was performed by affinity chromatography using a 5-ml HiTrap protein A HP column following manufacturer’s instructions (GE Healthcare, Piscataway, NJ). Purified IgG protein concentration was determined by Nanodrop ND-1000 spectrophotometer. To test its blocking activity, 5 μg of purified rabbit anti-LuloHya IgG or rabbit pre-immune IgG as a control, were incubated with either LuloHya (10 nM final concentration) or SGE (1 pair of SG) for 30 min at 37°C. Hyaluronidase activity was measured by the hyaluronidase turbidimetric method described above.

### Western blot

Western blot was carried out as described elsewhere [9]. Briefly, the content of 5 *Lu*. *longipalpis* SG and 100 ng of LuloHya were separated by NuPAGE protein gels. Proteins were transferred to a nitrocellulose membrane (iBlot, Invitrogene) that was blocked overnight at 4°C with blocking buffer: 50 mM Tris, 150 mM NaCl containing 5% (w/v) powdered nonfat blotting-grade milk (Bio-Rad) and 0.05% of Tween-20 (Sigma). Mouse anti-LuloHya antibodies were diluted in blocking buffer (1:5,000) and incubated for 90 min. Goat anti-mouse conjugated to alkaline phosphatase (Sigma) diluted in blocking buffer (1:10,000) was used as a secondary antibody. Immunogenic protein bands were visualized by Western Blue Stabilized Substrate for Alkaline Phosphatase (Promega, Madison, WI) and reaction was stopped with distilled water.

### RT-PCR

Samples of 10 head and thorax were collected from female and male sand fly adults and kept in Trizol reagent (Life Technologies) at -80°C until used. Total RNA, conversion to cDNA and PCR protocols were performed as described before [59]. Specific primers to target LuloHya gene were designed (HyaP1F: 5’-ATTGGAAGGACGCTAAATGG-3’ and HyaP1R: 5’-CTTTCATTTCTTGCATTGGC-3’). As a reference gene, *Lu*. *longipalpis* S60 gene was amplified with specific primers (LuloS60F: 5’-CCTCGTTGATGATGATGTGG-3’ and LuloS60R: 5’-CGAAGAGTCCAGGCCAGTAG-3’). PCR products were visualized under UV light in a 1.2% agarose gel (E-gel, Invitrogen).

### Vertebrate blood feeding assays on mice

To test the blocking activity of the anti-LuloHya *in vivo*, a blood feeding experiment was carried out. Briefly, C57BL/6 mice were intraperitoneally injected with either 100 μg of rabbit anti-LuloHya IgG or rabbit naïve IgG (Sigma) as a control group. After 15 minutes, each mouse was anesthetized and placed on top of a cage containing five-day-old female sand flies that had been deprived of water and sugar the day before the experiment. *Lutzomyia longipalpis* female adults were allowed to feed on the animals for 12 min at 27°C in the dark. Sand flies were scored under a stereo microscope as unfed or blood fed. In order to investigate whether vaccination with the salivary proteins may lead to a modified blood feeding success rate with epidemiological implications, a set of experiments on second blood feedings were carried out. Fully engorged sand flies were separated from a first blood feeding, as previously described. The following day they were transferred to oviposition pots. Six days after the first blood feeding, sand flies were allowed to feed on a new passively immunized animal for 12 min at 27°C in the dark. Right after the second feeding, sand flies were scored as unfed or blood fed.

### Blood meal size

Blood fed sand flies were individually homogenized in 100 μl of PBS and cleared in a centrifuge for 5 min at 13,000 x *g*. Drabkin’s reagent (Sigma) was used to determine the hemoglobin content following the manufacturer's protocol with minor modifications. Each sample (40 μl of supernatant from a homogenized sand fly) was incubated for 15 min with 100 μl of Drabkin’s reagent in flat-bottom 96-well microtiter plates (Corning, Costar). Samples were tested in duplicates and absorbance at 540 nm was determined using a VersaMax microplate reader (Molecular Devices).

### Oviposition experiments

Five-day-old *Lu*. *longipalpis* females were blood fed on passively immunized mice with 100 μg of rabbit anti-LuloHya, anti-Lundep and naïve IgG as described before for at least 45 min to maximize the number of fully engorged sand flies. Three to four hours after feeding, fully blood fed females were selected under the stereo microscope and individually separated into 4 cm height x 2 cm diameter plastic vials filled with 1 cm of plaster of Paris. Sand flies were kept at 27°C with sugar *ad libitum* and allowed to lay the eggs. After 6 days, eggs were counted under a stereo microscope. Sand flies that died before laying eggs were excluded from the counting. Two independent experiments were carried out.

### *Leishmania major* parasites preparation

*Leishmania major* parasites (WR 2885 strain, Walter Reed Army Institute of Research) were cultured in Schneider’s medium supplemented with 10% heat-inactivated fetal bovine serum, 2 mM L-glutamine, 100 U/ml penicillin, and 100 μg/ml streptomycin. Metacyclic promastigotes were negatively isolated from stationary cultures with peanut agglutinin (Vector Laboratories, Inc., Burlingame, CT). Parasites were resuspended in phosphate saline buffer and counted in a Neubauer chamber.

### Vaccination experiments

Five-week-old mice of two different strains were used. C57BL/6 mice were purchased from Charles River Laboratories (Wilmington, MA) while the B-cell-deficient B6.129S2-*Ighm*^*tm1Cgn*^/J mice came from Jackson Laboratory (Bar Harbor, ME).

Mice were IM immunized with 10 μg of LuloHya, Lundep [9] or PBS as the control group in combination with Magic Mouse Adjuvant (CD Creative Diagnostics, Shirley, NY). Mice were boosted 3 weeks after the first immunization. Ten days later and coincident with the antibody production peak, mice were challenged with *L*. *major* metacyclic parasites. Both mice ears were intradermally inoculated with 10 μl containing 1,000 parasites and the content of one salivary gland from adult female *Lu*. *longipalpis* using a 29-gauge needle. Ear lesions were weekly monitored during 8 weeks by measuring ear thickness with a Vernier caliper (Mitutoya America Corporation, Aurora, IL). Parasite load in the ears was determined by limiting dilution assay as described elsewhere [9]. Briefly, homogenized ears were four-fold serially diluted with supplemented Schneider’s medium in microtiter culture plates. After 7 days of incubation at 27°C, wells were examined for motile promastigote presence under an inverted microscope. Parasite burden was expressed as the number of parasites per ear, considering that the last positive dilution contained at least one living parasite.

### ELISA

Cross reactivity between antibodies against LuloHya or Lundep and *L*. *major* was determined by ELISA according to Chagas *et al* [59] with minor modifications. Briefly, microtiter flat-bottom plates (Maxisorp, Nunc, Roskilde, Denmark) were coated with 100 ng of recombinant proteins (LuloHya or Lundep) and 1, 2 and 5 μg of *L*. *major* extract per well in carbonate-bicarbonate buffer pH 9.5 (Sigma) at 4°C for 16 h. *Leishmania major* extract was obtained by disrupting the cultured promastigotes by 3 cycles of freezing and thawing with liquid nitrogen and a 60°C water bath. Plates were blocked with 5% BSA in TBS (25 mM Tris, 150 mM NaCl, pH 7.4) for 2 h at room temperature. After 3 washes with TBS supplemented with 0.05% (v/v) Tween (TTBS) primary antibodies (mouse anti-LuloHya and mouse anti-Lundep [9], both diluted at 1:1,000 in TTBS) were added. Mouse anti-tubulin, beta, clone KMX-1 (MAB3408, lot 2452493, EMD Millipore), 1:2,000 in TTBS, was used as a positive control for parasite recognition. After 1 h incubation and further washing, alkaline phosphatase-coupled anti-mouse IgG (1:10,000 in TTBS, Sigma) was added. Following another washing cycle, plates were developed with stabilized p-nitrophenyl phosphate (Sigma) and absorbance was measured at 405 nm in a VersaMax microplate reader (Molecular Devices) after a 15-min incubation.

The amount of LuloHya in the salivary glands of *Lu*. *longipalpis* was assessed by ELISA, as previously described. Plates were coated with a serial dilution of recombinant LuloHya protein (50–0.78 ng) to generate a standard curve which was used to infer the amount of LuloHya in *Lu*. *longipalpis* SGE obtained from 10 and 5 salivary glands in two independent experiments (linear fit, R^2^ = 0.9909).

### Fluorescence microscopy

*Leishmania major* promastigotes from a stationary culture were collected and fixed in 4% paraformaldehyde (Sigma). Immediately, 200 μl of 10^7^ parasites/ml were dispensed into each well of an 8-well chamber slides (Lab-Tek, Nunc, Thermo Fisher Scientific) and incubated for 24 h at 4°C. After 3 washes of 10 min with PBS, slides were blocked with 200 μl of 1% BSA, 0.5% Triton X-100 in PBS (blocking buffer) for 30 min. Mouse anti-LuloHya, anti-Lundep (1:1,000) and anti-tubulin, beta, clone KMX-1 (EMD Millipore, 1:2,000) were diluted in blocking buffer and incubated at 4°C for 16 h. After 3 washes with blocking buffer, samples were incubated with 10 μg/ml rabbit anti-mouse IgG conjugated with Alexa Fluor 488 (Life Technologies) diluted in 0.05% Tween, PBS (v/v) for 1 h. Excess of conjugate was removed by 3 additional washes with blocking buffer and slides were mounted with ProLong Gold Antifade Mountant with DAPI (Invitrogen). Differential Interference Contrast (DIC) and fluorescent images were acquired in a Leica EpiFluorescence Microscope, using an oil immersion 100X objective with a 1.6X digital magnification.

### Cell culture and cytokine expression pattern

A primary culture of HMVEC was obtained from Lonza. Cells were grown at 37°C in a 5% CO_2_ incubator with Endothelial Cell Basal Medium-2 (EBM-2, Clonetics, Lonza), supplemented with EGM^TM^-2 Single Quote (Lonza) and subcultured using the Clonetics ReagentPack (Lonza). The day before an experiment, cells (in their fourth passage) were detached and distributed into a 12-well culture plate (5x10e^5^ cells/well). Complete medium was removed and replaced by incomplete medium (EBM-2 without growth factors) for 4 h. Starved cells were incubated with LuloHya, Lundep (both at a final concentration of 1 μM) or SGE of 10 pairs of *Lu*. *longipalpis* SG for another 4 h. Control wells were incubated with only incomplete medium. Cells were collected in Trizol reagent (Invitrogen) and total RNA isolation and cDNA conversion was prepared as described before [58]. Cytokine expression pattern was assessed using The Human Cytokines & Chemokines RT^2^ Profiler PCR Array PAHS-150ZD (Qiagen, Valencia, CA) that includes expression profiles of 84 key secreted proteins for immune response and other functions. Data analysis was carried out with RT^2^ Profiler PCR Array Data Analysis version 3.5 following the software guidelines (SABioscience, Qiagen, https://www.qiagen.com/us/shop/genes-and-pathways/data-analysis-center-overview-page/). Parameters were as follows: Ct cut-off was set at 35 cycles. All RT-PCR data were adjusted to the same threshold. After checking stable amplification of positive controls for all samples and absence of genomic DNA contamination results were normalized against the housekeeping genes HPRT1 (Refseq No. NM_000194) and RPLP0 (Refseq No. NM_001002). Only genes with a fold change greater than 4 were considered. Expression levels of the cytokines CSF2, CSF3, CXCL1, CXCL2, CXCL8 and LIF were further validated by qPCR with specific primers (Qiagen, Valencia, CA). HPRT1 was chosen as a reference gene. qPCR was performed as described before [58].

### Venom serine protease (HF) isolation and characterization

Isolation of the serine protease was achieved by cation exchange chromatography. Venom sample (5 mg) from *Crotalus oreganus helleri* (Southern Pacific rattlesnake) were dissolved in 200 μL of 20 mM Tris-HCl pH 8.0 buffer and injected into a cationic exchange column (Sulfopropyl Waters Protein Pak 7.5 x 75 mm-10 μm, Milford, MA) equilibrated with 20 mM Tris-HCl, pH 8.0 buffer at a 1 mL/min flow rate. The eluting buffer was integrated linearly from 0 to 100% using a 20 mM Tris-HCl, pH 8.0 buffer containing 0.5 M NaCl. The proteins were eluted at a 1 mL/min flow rate over 90 min using a Waters 1525 binary HPLC system (Milford, MI, USA). A Waters 2487 dual λ absorbance detector (Milford, MI, USA) was used to monitor absorbance at 280 nm and Waters Breeze software was used to control the HPLC system and store the data.

Four μg of purified venom protein was transferred from an SDS-PAGE onto a polyvinylidene difluoride (PVDF) membrane (Millipore Corporation, MA, USA) using a Trans-Blot SD semi-dry transfer cell (Bio-Rad, USA) at 125 mA for 1 h. The membrane was stained with Coomassie blue R-250 for 5 min and destained with 50% methanol for 5 min. A target band was excised from the membrane and subjected to N-terminal sequence analysis using a PPSQ-33B protein sequencer (SHIMADZU, Kyoto, Japan) following the manufacturer’s instructions. Sequencing was performed for 14 cycles. The 14 N-terminal amino acid sequences were compared to the sequences in the GenBank database using GenBank BLASTP programs.

### Dermonecrotic assay

A dermonecrotic assay was used to assess hemorrhage and edema induced by SGE and recombinant salivary proteins. SGE of two pairs of *Lu*. *longipalpis* SG, 10 μg of LuloHya or Lundep and a combination of 5 μg of each recombinant protein were injected intradermally into the ears of BALB/c mice along with 3 μg of venom serine protease (HF) or PBS as control. After 2 h, ears were excised and dermonecrotic lesions were recorded. Ears were formalin-fixed in our laboratory and histological preparation (paraffin inclusion, sectioning and hematoxylin/eosin staining) were processed by Histoserv Inc. (Germantown, MD). Examination of histological samples were examined under the microscope at the Infectious Disease Pathogenesis Section (NIAID, NIH).

### Flow cytometry analysis of neutrophil recruitment

C57BL/6 mice ears were intradermally injected with LuloHya (10 μg and 1 μg), Lundep (10 μg and 1 μg), *Lu*. *longipalpis* SGE (equivalent to 2 pairs of salivary glands). As negative controls, ears were injected either with PBS or with a non-related salivary protein from the mosquito *Ae*. *aegypti* which was expressed and purified in the same manner. After 2 h, mice were euthanized, and the two sheets of ear dermis were separated, deposited in PBS containing 0.2 mg/ml Liberase CI purified enzyme blend (Roche Diagnostics Corp.), and incubated for 1 h at 37°C. Digested tissue was placed in a grinder and processed in a tissue homogenizer (Medimachine; Becton Dickenson). Tissue homogenates were filtered using a 30 μm Filcon filters (BD). The resulting single cell suspensions were first stained with the Fixable Yellow Dead Cell Stain Kit (Invitrogen) for 20 min. The suspension was then washed and incubated with anti-Fc (CD16/32) antibodies to block non-specific binding. After 10 min, the cells were stained for Ly6C (clone AL-21; FITC; BD), Ly6G (clone 1A8; PE; BD) and CD11b (clone M1/70; PE-Cy7; BD) for 30 min. Cells were gated based on forward scatter and side scatter parameters and further gated on live cells. Cells were acquired on a MACSQuant flow cytometer (Miltenyi Biotec) and data were analyzed with FlowJo Software 4.3.

### Statistical analysis

GraphPad Prism v 7.01 (GraphPad Software, Inc., San Diego, CA) was used to analyze data. Comparisons between study groups were determined with a 2-tailed *t* test with 2-way analysis of variance and 95% confidence interval. Statistical significance was set as p value <0.05.

## Supporting information

S1 Fig Antibodies against LuloHya and Lundep do not recognize *L*. *major* parasites. **(A)** IgG antibody levels of mouse anti-LuloHya and mouse anti-Lundep sera when using 100 ng of either LuloHya or Lundep or 5 μg of *L*. *major* extract per well as antigens. As a positive control of the *L*. *major* extract, mouse anti-tubulin antibody was used. Antibody levels are expressed as the mean of the adjusted absorbance of technical triplicates at 405 nm (Abs_405_) ± SEM. Multiple comparisons were done by one-way ANOVA (****: p<0.0001). **(B)** Fluorescence microscopy of *L*. *major* promastigotes incubated with mouse anti-LuloHya, anti-Lundep. As a positive control, fixed promastigotes were incubated with mouse anti-tubulin and as negative control, no serum was added. Images were taken at 100X magnification. DIC: Differential Interference Contrast. AF 488: (Alexa Fluor 488). Scale bar: 10 μm.
(TIF)

S2 Fig Relevance of LuloHya and Lundep on sand fly blood feeding and other physiological parameters. **(A)** Circulating rabbit anti-LuloHya antibodies in mice significantly reduced the feeding success of sand flies on passively immunized mice when compared to the sand flies fed on mice immunized with rabbit pre-immune IgG (IgG-Control). Fed: number of blood fed female sand flies recorded under the stereoscope. UF: Number of unfed female sand flies, expressed in percentages. Graph represents data from 3 independent experiments (average of 340 sand flies per group). Results were analyzed using a χ2 test. **(B)** No significant differences in blood meal size ingested by sand flies that fed on mice was revealed by measuring the total hemoglobin content in the midguts using Drabkin’s reagent. Results are expressed as the absorbance at 540 nm (number of individual engorged sand flies analysed = 28). **(C)** Number of laid eggs by sand fly females fed to repletion on mice passively immunized with rabbit anti-LuloHya and anti-Lundep. As control, oviposition data of *Lu*. *longipalpis* fed on mice injected with rabbit pre-immune IgG was recorded. Multiple comparisons done by one-way ANOVA showed no differences in the oviposition rate (data from 2 independent experiments, average of 58 sand flies per group).
(TIF)

S3 Fig Endonuclease in *Phlebotomus* spp. Endonuclease activity is present in *P*. *papatasi* and *P*. *duboscqi* salivary glands. Plasmid DNA (200 ng) was incubated in a 15 μl final volume with 5–7 day old female SGE (the equivalent of 1 salivary gland pair). After 10 min at 37°C, samples were electrophoresed in a 1.2% e-gel and visualized under UV light. Lane 1: *P*. *duboscqi*, Lane 2: *P*. *papatasi* (Saudi Arabia), Lane 3: *P*. *papatasi* (Turkey), Lane 4: Dnase-I (0.5 U), Lane 5: Negative control.
(TIF)
