# Supplementary figures and images for: Immunity to LuloHya and Lundep, the salivary spreading factors from Lutzomyia longipalpis, protects against Leishmania major infection
Source: PLoS Pathog. 2018 May 3;14(5):e1007006. doi: 10.1371/journal.ppat.1007006 (PMC5953502; doi:10.1371/journal.ppat.1007006)

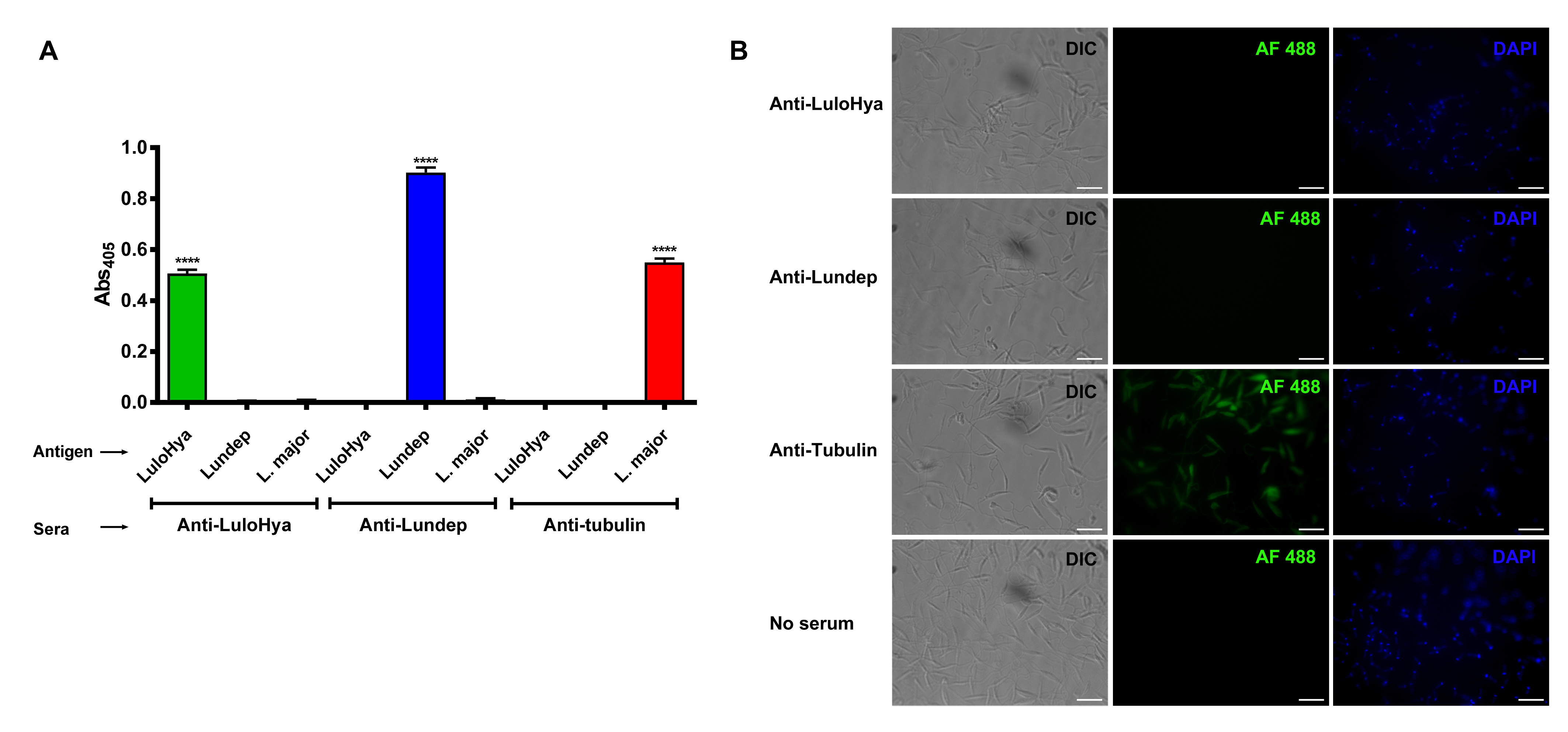

Supplement: S1 Fig — (A) IgG antibody levels of mouse anti-LuloHya and mouse anti-Lundep sera when using 100 ng of either LuloHya or Lundep or 5 μg of L. major extract per well as antigens. As a positive control of the L. major extract, mouse anti-tubulin antibody was used. Antibody levels are expressed as the mean of the adjusted absorbance of technical triplicates at 405 nm (Abs405) ± SEM. Multiple comparisons were done by one-way ANOVA (****: p<0.0001). (B) Fluorescence microscopy of L. major promastigotes incubated with mouse anti-LuloHya, anti-Lundep. As a positive control, fixed promastigotes were incubated with mouse anti-tubulin and as negative control, no serum was added. Images were taken at 100X magnification. DIC: Differential Interference Contrast. AF 488: (Alexa Fluor 488). Scale bar: 10 μm. (TIF) [file ppat.1007006.s001.tif]

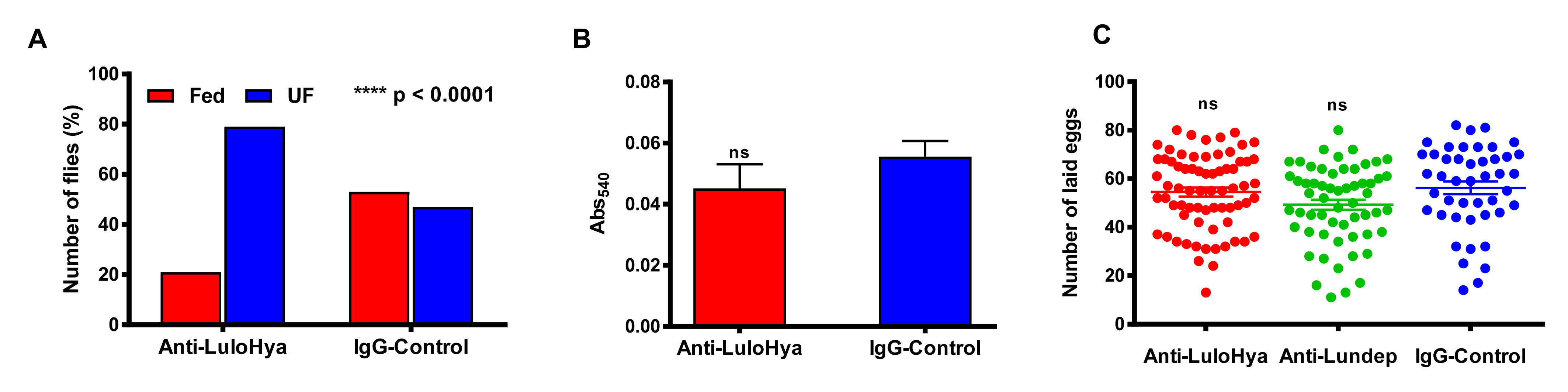

Supplement: S2 Fig — (A) Circulating rabbit anti-LuloHya antibodies in mice significantly reduced the feeding success of sand flies on passively immunized mice when compared to the sand flies fed on mice immunized with rabbit pre-immune IgG (IgG-Control). Fed: number of blood fed female sand flies recorded under the stereoscope. UF: Number of unfed female sand flies, expressed in percentages. Graph represents data from 3 independent experiments (average of 340 sand flies per group). Results were analyzed using a χ2 test. (B) No significant differences in blood meal size ingested by sand flies that fed on mice was revealed by measuring the total hemoglobin content in the midguts using Drabkin’s reagent. Results are expressed as the absorbance at 540 nm (number of individual engorged sand flies analysed = 28). (C) Number of laid eggs by sand fly females fed to repletion on mice passively immunized with rabbit anti-LuloHya and anti-Lundep. As control, oviposition data of Lu. longipalpis fed on mice injected with rabbit pre-immune IgG was recorded. Multiple comparisons done by one-way ANOVA showed no differences in the oviposition rate (data from 2 independent experiments, average of 58 sand flies per group). (TIF) [file ppat.1007006.s002.tif]

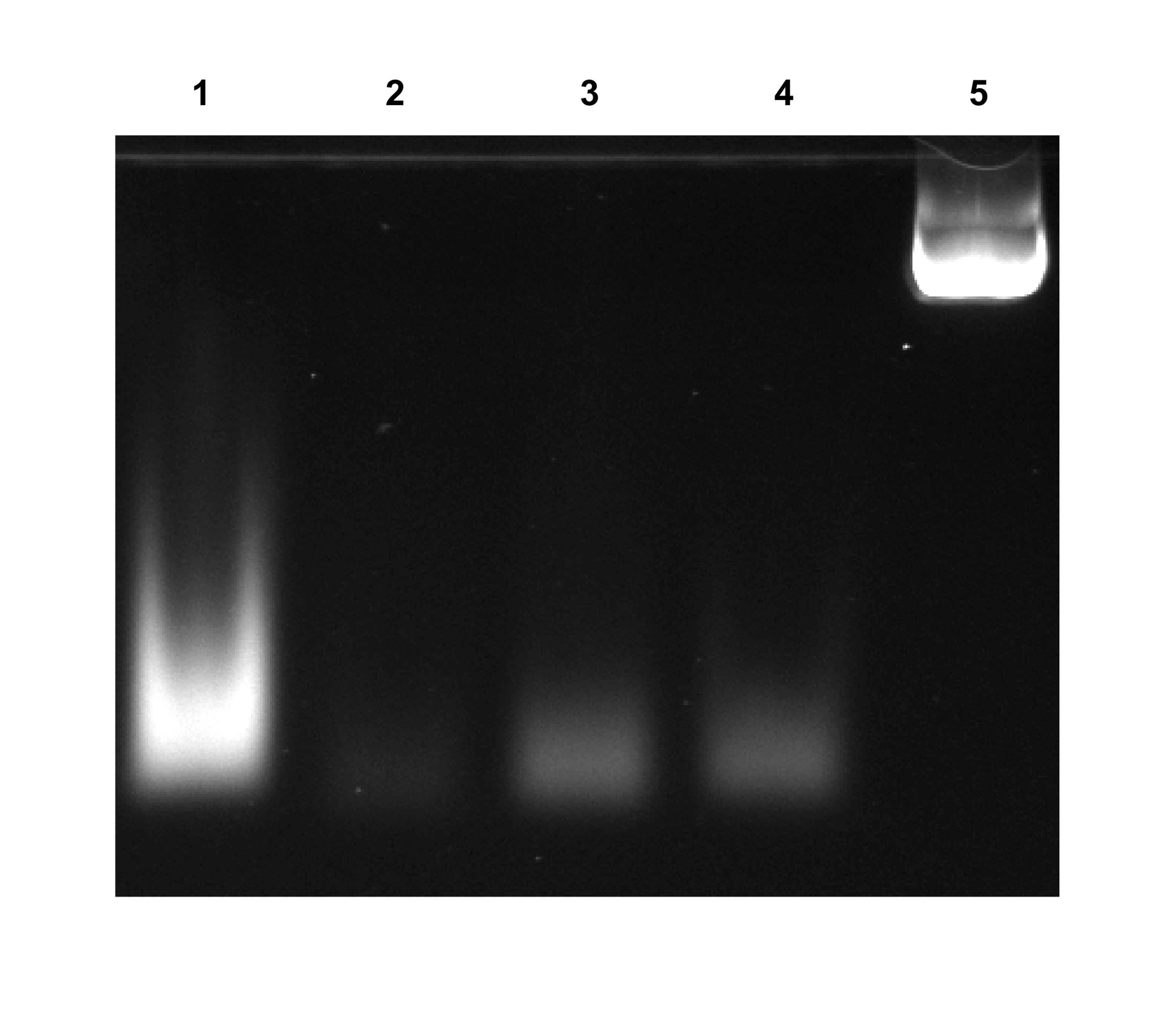

Supplement: S3 Fig — Endonuclease activity is present in P. papatasi and P. duboscqi salivary glands. Plasmid DNA (200 ng) was incubated in a 15 μl final volume with 5–7 day old female SGE (the equivalent of 1 salivary gland pair). After 10 min at 37°C, samples were electrophoresed in a 1.2% e-gel and visualized under UV light. Lane 1: P. duboscqi, Lane 2: P. papatasi (Saudi Arabia), Lane 3: P. papatasi (Turkey), Lane 4: Dnase-I (0.5 U), Lane 5: Negative control. (TIF) [file ppat.1007006.s003.tif]
